# Three Pairs of New Spirocyclic Alkaloid Enantiomers From the Marine-Derived Fungus *Eurotium* sp. SCSIO F452

**DOI:** 10.3389/fchem.2019.00350

**Published:** 2019-05-20

**Authors:** Weimao Zhong, Junfeng Wang, Xiaoyi Wei, Tingdan Fu, Yuchan Chen, Qi Zeng, Zhonghui Huang, Xinan Huang, Weimin Zhang, Si Zhang, Lijuan Long, Fazuo Wang

**Affiliations:** ^1^CAS Key Laboratory of Tropical Marine Bio-resources and Ecology, Guangdong Key Laboratory of Marine Materia Medica, RNAM Center for Marine Microbiology, South China Sea Institute of Oceanology, Chinese Academy of Sciences, Guangzhou, China; ^2^College of Earth and Planetary Sciences, University of Chinese Academy of Sciences, Beijing, China; ^3^Key Laboratory of Plant Resources Conservation and Sustainable Utilization, South China Botanical Garden, Chinese Academy of Sciences, Guangzhou, China; ^4^Institute of Tropical Medicine, Guangzhou University of Chinese Medicine, Guangzhou, China; ^5^State Key Laboratory of Applied Microbiology Southern China, Guangdong Provincial Key Laboratory of Microbial Culture Collection and Application, Guangdong Open Laboratory of Applied Microbiology, Guangdong Institute of Microbiology, Guangzhou, China

**Keywords:** *Eurotium* sp. SCSIO F452, spirocyclic alkaloid enantiomers, antioxidative and cytotoxic activities, Diels-Alder cycloaddition, molecular docking

## Abstract

Three pairs of new spirocyclic alkaloid enantiomers eurotinoids A–C (**1**–**3**), as well as a known biogenetically related racemate dihydrocryptoechinulin D (**4**) were isolated from a marine-derived fungus *Eurotium* sp. SCSIO F452. Their structures were determined by spectroscopic analyses and electronic circular dichroism (ECD) calculations. Compounds **1** and **2** represent the first two “*meta*” products from a non-stereoselective [4 + 2] Diels-Alder cycloaddition presumably between an enone group of a diketopiperazine alkaloid and a diene group of a benzaldehyde derivative via a new head-to-tail coupling mode biosynthetically, while **3** and **4** were “*ortho*” products. Their enantiomers exhibited different antioxidative and cytotoxic activities. The modes of action were investigated by a preliminary molecular docking study.

## Introduction

Marine fungi are widely known as a rich source producing structurally diverse and biologically active natural products, which attract people's great interests in pharmaceutical, agrochemical, and food industries (Richards et al., 2012; Martins et al., 2014; Blunt et al., 2018). The fungal genus *Eurotium*, known as the teleomorph of *Aspergillus*, has been found to produce diketopiperazine (DKP) alkaloids, prenyl benzaldehyde derivatives, anthraquinones, and so on, which display a wide range of biological activities such as antibacterial, antioxidative, insecticidal, and binding with opioid and cannabinoid receptors activities (Gao et al., 2011; Du et al., 2012, 2017; Gomes et al., 2012; Meng et al., 2015). Motivated by the fascinating secondary metabolites produced by the fungi *Eurotium* spp., we have launched a chemical investigation on a marine-derived fungus isolated from a South China Sea sediment sample, *Eurotium* sp. SCSIO F452, and found a series of prenylated DKPs, prenyl benzaldehyde derivatives, especially three pairs of spirocyclic diketopiperazine-anthraquinone enantiomers variecolortins A–C (Wang et al., 2013; Zhong W. et al., 2018; Zhong W.-M. et al., 2018). In order to enrich and improve the chemical diversity of this fungus, we carried out a further chemical study on the *Eurotium* sp. SCSIO F452, and disclosed three pairs of new spirocyclic alkaloid enantiomers eurotinoids A–C (**1**–**3**), as well as a known biogenetically related racemate dihydrocryptoechinulin D (**4**) (Gao et al., 2012). Structurally, compounds **1**–**4** feature a cyclohexene core presumably deriving from a key non-stereoselective [4 + 2] Diels-Alder cycloaddition between an enone group of a diketopiperazine alkaloid and a diene group of a benzaldehyde derivative. Interestingly, compounds **1** and **2**, **3** and **4** occurred as two pairs of diastereomers. The molecular structures of all the compounds including absolute configurations were unambiguously assigned by comprehensive spectroscopic data and quantum chemical electronic circular dichroism (ECD) calculations. Herein we report the isolation, structure elucidation, plausible biosynthetic pathway, antioxidative and cytotoxic bioactivities, and molecular dockings of **1**–**4**.

## Materials and Methods

### General Experimental Procedures

Optical rotations were measured with an MCP 500 automatic polarimeter (Anton Paar) with MeCN as solvent. UV spectra were recorded on a UV-2600 spectrometer (Shimadzu). IR spectra were measured on an IR Affinity-1 spectrometer (Shimadzu). ^1^H, ^13^C NMR, DEPT, and 2D NMR spectra were recorded on the AVANCE III HD 700 (Bruker). Circular dichroism spectra were measured with a Chirascan circular dichroism spectrometer (Applied Photophysics). HRESIMS spectra data were recorded on a MaXis quadrupole-time-of-flight mass spectrometer. Thin layer chromatography (TLC) was performed on plates precoated with silica gel GF_254_ (10–40 μm). Column chromatography (CC) was performed over silica gel (200–300 mesh and 300–400 mesh) (Qingdao Marine Chemical Factory) and octadecylsilyl silica gel (ODS) (50 μm, YMC). High performance liquid chromatography was performed on an Agilent 1260 HPLC equipped with a DAD detector, using an ODS column (YMC-pack ODS-A, 10 × 250 mm, 5 μm), two chiral-phase columns (Daicel chiralpak IA and IC, 4.6 × 250 mm, 5 μm). All solvents used in CC and HPLC were of analytical grade (Tianjin Damao Chemical Plant, Tianjin, China) and chromatographic grade (Oceanpak, Sweden), respectively. Fractions were monitored by TLC and spots were visualized by heating silica gel plates sprayed with 10% H_2_SO_4_ in EtOH.

### Fungal Material

The fungal strain used in this investigation was isolated from a South China Sea sediment sample (17°29.804'N, 110°0.292'E) at a depth of 158 m in May, 2010. It was identified as *Eurotium* sp. SCSIO F452 according to a molecular biological protocol by DNA amplification and sequencing of the ITS region (deposited in GenBank, accession no. JX481973). The working strain was prepared on potato dextrose agar slants modified with seawater instead of distilled water and stored at 4 °C. A reference culture was maintained at −80 °C in RNAM Centre for Marine Microbiology, South China Sea Institute of Oceanology, Chinese Academy of Sciences.

### Fermentation and Extraction

The fungus *Eurotium* sp. SCSIO F452 was cultured and extracted as previously described (Zhong W.-M. et al., 2018).

### Isolation and Purification

The EtOAc extract (68 g) was subjected to vacuum liquid chromatography (VLC) on a silica gel column using step gradient elution with petroleum ether (PE)/EtOAc (1:0 to 0:1) and CHCl_3_/MeOH (1:0 to 0:1) to separate into 18 fractions based on TLC properties. Fr.3 (10 g) was separated by silica gel CC (PE/Acetone 1:0 to 0:1) to obtain 10 subfractions (Frs.3.1–3.10). Then Fr.3.9 (0.8 g) was divided into seven parts (Frs.3.9.1–3.9.7) by ODS CC with a gradient elution of MeOH/H_2_O (7:3 to 1:0). Fr.3.9.3 (56 mg) was further purified by repeated semi-preparative HPLC (3 mL/min, 59% CH_3_CN/H_2_O) to yield **1** (3.1 mg), **2** (1.5 mg), **3** (2.0 mg), and **4** (4.2 mg).

### Chiral Separation of Enantiomers

Compounds **1**–**4** were subjected to chiral-phase HPLC separation on an Agilent 1260 liquid chromatograph system equipped with a DAD detector. Compounds (+)-**1** (1.3 mg, t_R_ = 10.825 min) and (–)-**1** (1.4 mg, t_R_ = 23.026 min), (+)-**3** (0.8 mg, t_R_ = 11.887 min) and (–)-**3** (0.8 mg, t_R_ = 17.220 min) were separated by a Daicel chiralpak IC column (250 × 4.6 mm, 5 μm), using an elution mixture of n-hexane/isopropanol (87:13) at a flow rate of 1 mL/min. Compounds (+)-**2** (0.6 mg, t_R_ = 6.379 min) and (–)-**2** (0.6 mg, t_R_ = 7.053 min) were separated by a Daicel chiralpak IA column (250 × 4.6 mm, 5 μm), using n-hexane/isopropanol (72:28) mixture elution at a flow rate of 0.9 mL/min. Compounds (+)-**4** (1.8 mg, t_R_ = 22.237 min) and (–)-**4** (1.7 mg, t_R_ = 34.315 min) were separated by a Daicel chiralpak IC column (250 × 4.6 mm, 5 μm), using an elution mixture of n-hexane/isopropanol (90:10) at a flow rate of 1 mL/min.

### Computational Details

Molecular Merck force field (MMFF) calculations were done using Spartan'14 program (Wavefunction Inc., Irvine, CA, USA). Density functional theory (DFT) and time-dependent density functional theory (TDDFT) calculations were performed with Gaussian09 program package (Frisch et al., 2010). In order to reduce the computational cost, the truncated structures (Figure S5) were used in ECD calculations of compounds (12*S*,28*R*,31*R*)-**1**′, (12*S*,28*S*,31*S*)-**2**′, and (12*R*,28*R*,31*R*)-**3**′, corresponding to innate compounds (12*S*,28*R*,31*R*)-**1**, (12*S*,28*S*,31*S*)-**2**, and (12*R*,28*R*,31*R*)-**3**. For conformational analysis, the conformers generated by a MMFF conformational search in an energy window of 10 kcal/mol were subjected to geometry optimization using the DFT method at the B3LYP/def2-SVP level (Lee et al., 1988; Becke, 1993; Weigend and Ahlrichs, 2005). Frequency calculations were run at the same level to estimate their relative thermal (Δ*E*) and free energies (Δ*G*) at 298.15 K. Energies of the low-energy conformers in MeCN were re-calculated at the M06-2X/def2-TZVP level. Solvent (MeCN) effects were taken into account by using polarizable continuum model (IEFPCM). The TDDFT calculations were performed using the hybrid PBE1PBE (Perdew et al., 1996; Adamo and Barone, 1999) and M06-2X (Zhao and Truhlar, 2008) functionals, and the Ahlrichs' basis sets TZVP (Schäfer et al., 1994) and/or def2-SVP. The number of excited states is 36 for each of the compound. The ECD spectra were generated by the program SpecDis (Bruhn et al., 2013) using a Gaussian band shape from dipole-length dipolar and rotational strengths. The equilibrium population of each conformer at 298.15 K was calculated from its Δ*G* using Boltzmann statistics. The calculated spectra of compounds were generated from the low-energy conformers according to the Boltzmann weighting of each conformer in MeCN solution.

### Biological Assays

The details of the experimental procedures for antioxidative and cytotoxic bioassays were similar to those presented in our former paper (Zhong W.-M. et al., 2018).

### Molecular Docking Study

The detail procedures of molecular docking study were presented in Supporting Information File.

## Results and Discussion

### Identification of Compounds 1**–**3

**Figure 1:**
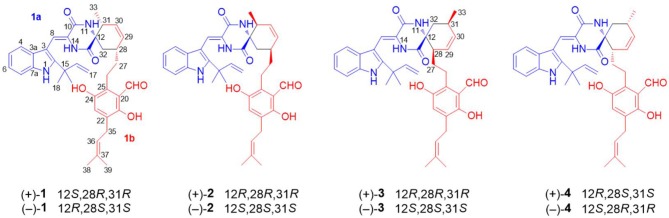
Structures of enantiomers **1**–**4**.

A 30 L fermentation broth of the marine-derived fungus *Eurotium* sp. SCSIO F452 furnished a crude extract (68 g), which was subsequently separated by repeated chromatographic methods including column chromatography (CC) over silica gel, ODS, and finally semi-preparative HPLC to yield compounds **1**–**4** (Figure 1). Eurotinoid A (**1**) was isolated as a yellow solid. Its molecular formula was determined as C_38_H_43_N_3_O_5_ by the positive HRESIMS (*m/z* 622.3270 [M + H]^+^, calcd for 622.3275), indicating 19 degrees of unsaturation. Its IR absorptions suggested the presence of hydroxyl and amine groups (3350, 3337 cm^−1^) and carbonyl functionalities (1682, 1636 cm^−1^). The ^1^H NMR spectrum of **1** (Table 1) recorded in CD_3_COCD_3_ showed five methyls at δ_H_ 1.07 (d, *J* = 7.2 Hz), 1.56 (s), 1.59 (s), 1.67 (s), and 1.70 (s), 12 olefinic protons ranging from δ_H_ 5.08 to 7.40, and six exchangeable protons at δ_H_ 6.53 (s), 8.04 (s), 8.21 (s), 10.36 (2H, overlap), and 11.98 (s). The ^13^C NMR and DEPT revealed the presence of 38 carbon resonances, including five methyls, five methylenes (one olefinic carbon), 12 methines (10 olefinic carbons), 16 non-protonated carbons (one nitrogenated carbon, three carbonyls, and nine olefinic carbons). By the aid of HSQC spectrum, all proton resonances were unambiguously assigned to their respective carbons except for the exchangeable protons.

**Table 1:** ^1^H and ^13^C NMR Data for **1**–**3** (700, 175 MHz, TMS, δ in ppm, *J* in Hz).

	**1**^**a**^		**2**^**a**^		**3**^**b**^	
**No**.	**δ_**C**_**	**δ_**H**_ (*J*, Hz)**	**δ_**C**_**	**δ_**H**_ (*J*, Hz)**	**δ_**C**_**	**δ_**H**_ (*J*, Hz)**
1		10.36 br s		10.30 br s		12.04 br s
2	145.1		145.1		144.3	
3	104.4		104.7		103.9	
3a	127.3		127.3		125.4	
4	119.7	7.26 d (7.8)	119.9	7.27 d (8.0)	119.2	7.16 d (7.9)
5	120.7	7.06 overlap	120.5	7.02 dt (7.5, 1.0)	119.2	6.86 dt (7.5, 0.8)
6	122.1	7.10 overlap	122.1	7.10 dt (7.5, 1.0)	120.6	7.04 dt (7.5, 0.8)
7	112.4	7.40 d (8.1)	112.4	7.40 d (8.1)	111.5	7.40 d (8.1)
7a	136.2		136.2		135.0	
8	111.4	7.11 s	111.7	7.09 s	111.3	7.05 s
9	126.1		126.8		124.4	
10	161.5		162.2		161.8	
11		6.53 s		6.91 s		7.93 s
12	61.2		61.1		59.6	
13	168.7		168.0		167.4	
14		8.04 s		8.16 s		9.01 s
15	40.0		40.1		39.0	
16	145.9	6.16 dd (17.4, 10.6)	146.0	6.16 dd (17.4, 10.6)	145.2	6.07 dd (17.6, 10.5)
17	112.4	5.11 dd (17.4, 0.8)	112.4	5.12 dd (17.4, 1.2)	111.7	5.02 dd (17.6, 1.1)
		5.08 dd (10.6, 0.8)		5.09 dd (10.6, 1.2)		5.05 dd (10.5, 1.1)
18	27.9	1.56 s	28.0	1.56 s	27.5	1.48 s
19	27.9	1.59 s	27.9	1.58 s	27.7	1.46 s
20	118.4		118.4		117.3	
21	155.5		155.5		153.3	
22	128.8		128.7		127.2	
23	126.5	7.06 s	126.4	7.07 s	125.4	7.00 s
24	147.8		147.7		146.9	
25	129.2		129.6		128.5	
26	22.9	3.14 m	21.6	3.13 m	20.9	a 2.96 m
						b 3.02 m
27	38.9	a1.91 m	38.7	1.77 m	35.3	a 1.48 overlap
		b1.84 m				b 1.74 overlap
28	34.5	2.59 m	32.2	2.71 m	40.0	2.44 m
29	131.1	5.95 dt (10.1, 2.9)	130.8	5.83 br d (10.1)	125.3	5.84 m
30	129.6	5.54 dt (10.1, 2.6)	129.7	5.77 dd (10.1, 4.7)	133.0	5.69 d (10.3)
31	36.7	2.96 overlap	41.8	2.57 m	29.0	2.60 m
32	38.5	2.30 dd (13.8, 8.2)	31.0	2.05 overlap	34.3	1.80 dd (13.9, 9.8)
		2.18 dd (13.8, 1.8)		1.97 dd (14.4, 6.3)		1.71 overlap
33	16.1	1.07 d (7.2)	17.8	1.15 d (7.0)	25.8	1.03 d (7.0)
34	197.4	10.36 s	197.4	10.37 s	197.1	10.22 s
35	27.6	3.22 d (7.3)	27.7	3.26 d (7.4)	26.7	3.19 d (7.4)
36	122.6	5.26 br t (7.3)	122.7	5.26 br t (7.4)	121.7	5.24 br t (7.4)
37	133.4		133.6		132.5	
38	25.8	1.70 s	25.9	1.72 s	25.6	1.70 s
39	17.7	1.67 s	17.8	1.69 s	17.6	1.66 s
OH-21		11.98 s		11.95 s		11.74 s
OH-24		8.21 s		8.10 s		9.09 s

a ***1** and **2** in Acetone-d_6_*.

b ***3** in DMSO-d_6_*.

**Figure 2:**
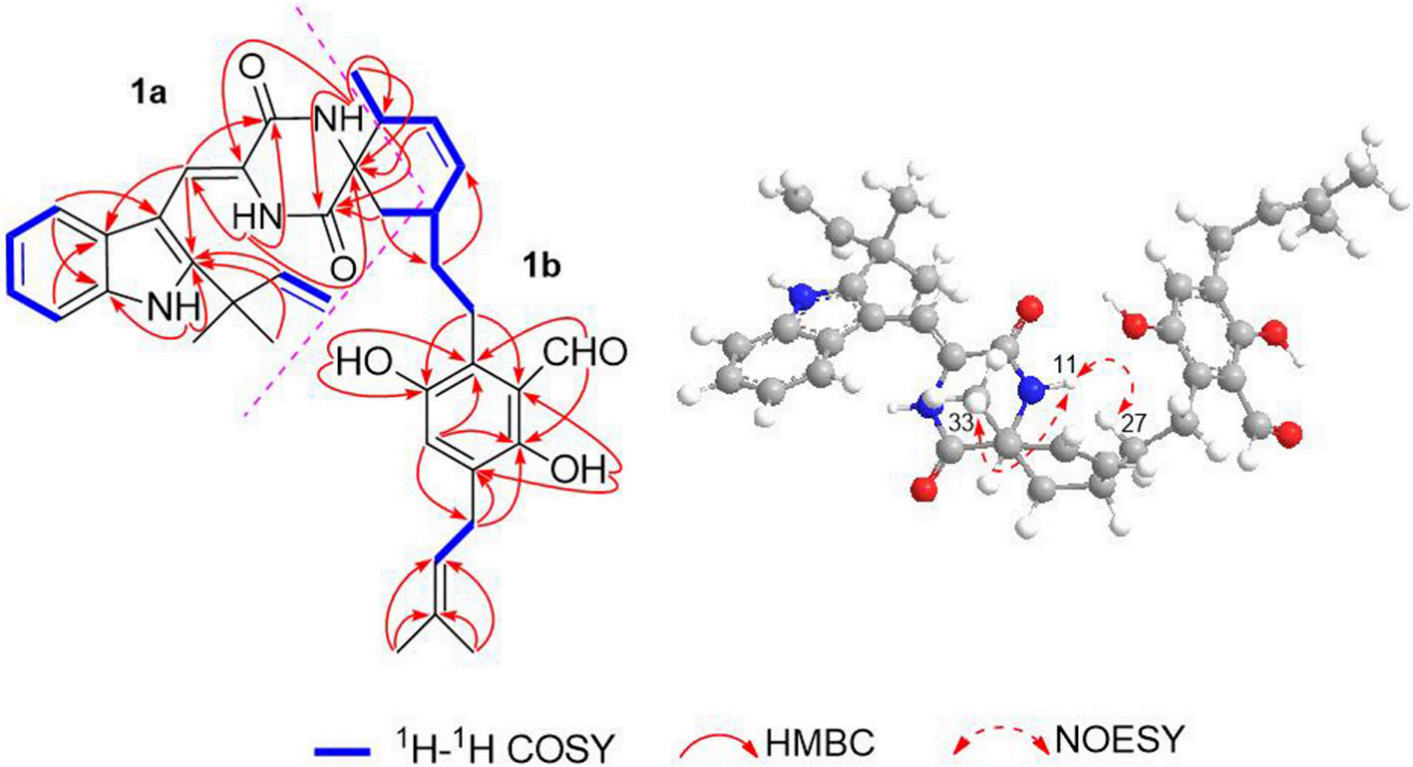
Key ^1^H–^1^H COZY, HMBC, and NOESY correlations of compound **1**.

The ^1^H–^1^H COZY spectrum of **1** established four spin-spin coupling systems shown in blue bold (Figure 2). The HMBC correlations between H-1 and C-2/C-3/C-7a, H-8 and C-3a/C-10, H-11 and C-9/C-13, H-14 and C-8/C-10/C-12, H-16/H_3_-18/H_3_-19, and C-2, H_2_-32 and C-12/C-13 implied the existence of a C-2 reverse prenylated indole DKP alkaloid unit (**1a**) in **1**. Moreover, the HMBC correlations between H-23 and C-21/C-25/C-35, H_2_-26 and C-20/C-24/C-28, H_2_-27 and C-29, H-29 and C-31, H-30 and C-28/C-33, H-34 and C-21/C-25, H_2_-35 and C-21/C-22/C-37, H_3_-38/H_3_-39 and C-36, OH-21 and C-20/C-22, OH-24 and C-24/C-25 supported the presence of a tetrasubstituted benzaldehyde derivative bearing a 3-methyl-2-butenyl and a C_7_-alkenyl side chain (**1b**). Extensive analysis of the 1D and 2D data demonstrated that **1** possessing two subunits **1a** and **1b**, shared partially structural similarities with dihydrocryptoechinulin D (**4**). However, careful analysis of its 2D NMR data revealed the two subunits were connected by a different coupling mode from **4**. The key ^1^H–^1^H COZY correlation of H_2_-32 and H-28, and HMBC correlations between H-11 and C-31, H-31/H_2_-32 and C-12/C-13, H_3_-33 and C-12 indicated that **1a** and **1b** were connected via C-12–C-31 and C-32–C-28 bonds forming a cyclohexene ring, rather than by C-12–C-28 and C-32–C-31 bonds in **4**. The diagnostic NOESY correlations (Figure 2) between H-11 and H_2_-27/H_3_-33 led to their assignment as α-orientation. The geometry of the Δ^8^ double bond was elucidated to be *Z* configuration by the downfield shift of H-8 (δ_H_ 7.11, s) due to the deshielding effect of the carbonyl group on the β-vinyl proton, which was also coincident with the lack of NOE effect between H-8 and H-14 (Gao et al., 2012).

**Figure 3:**
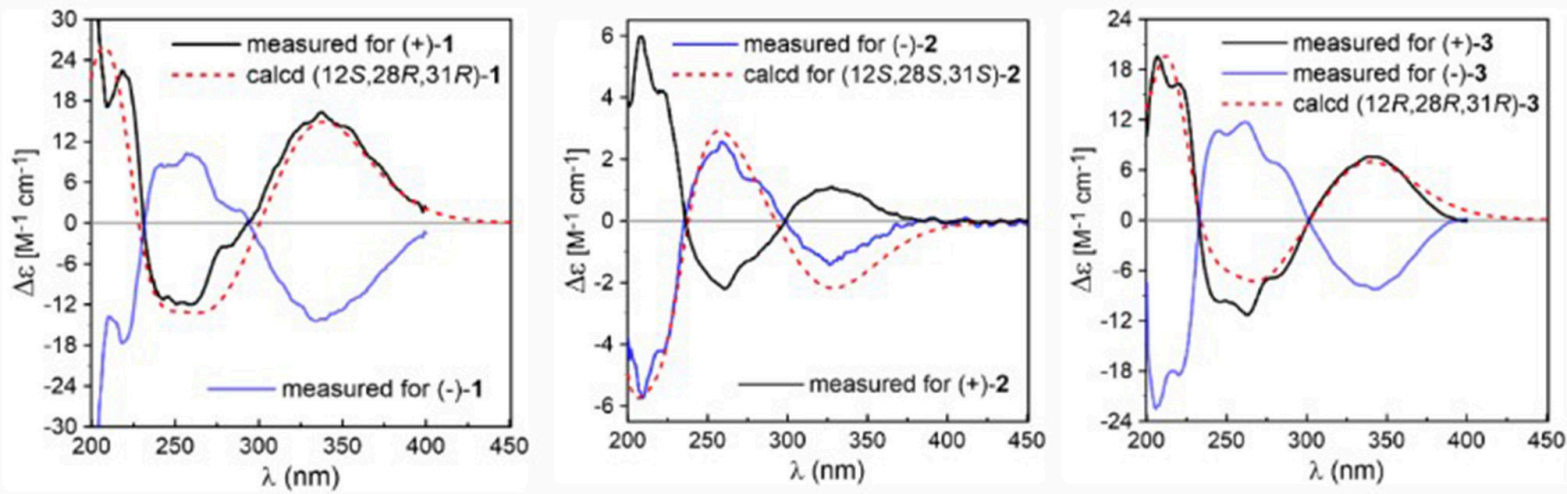
Comparison between experimental and calculated ECD spectra of **1**–**3** in MeCN.

Due to the baseline ECD curve and barely measurable specific rotation, compound **1** was assumed to be a racemic mixture, which was further confirmed by separation of its enantiomers by HPLC using a chiral column (Figure S1). The absolute configurations of individual (+)-**1** and (–)-**1** were determined by comparison of their calculated ECD spectra with experimental ones. As a result, the calculated ECD curve of 12*S*,28*R*,31*R*-**1** displayed good agreement with the experimental one of (+)-**1** (Figure 3), which could establish the absolute configurations of 12*S*,28*R*,31*R* for (+)-**1**, and therefore the 12*R*,28*S*,31*S* for (–)-**1** due to their enantiomeric nature.

**Figure 4:**
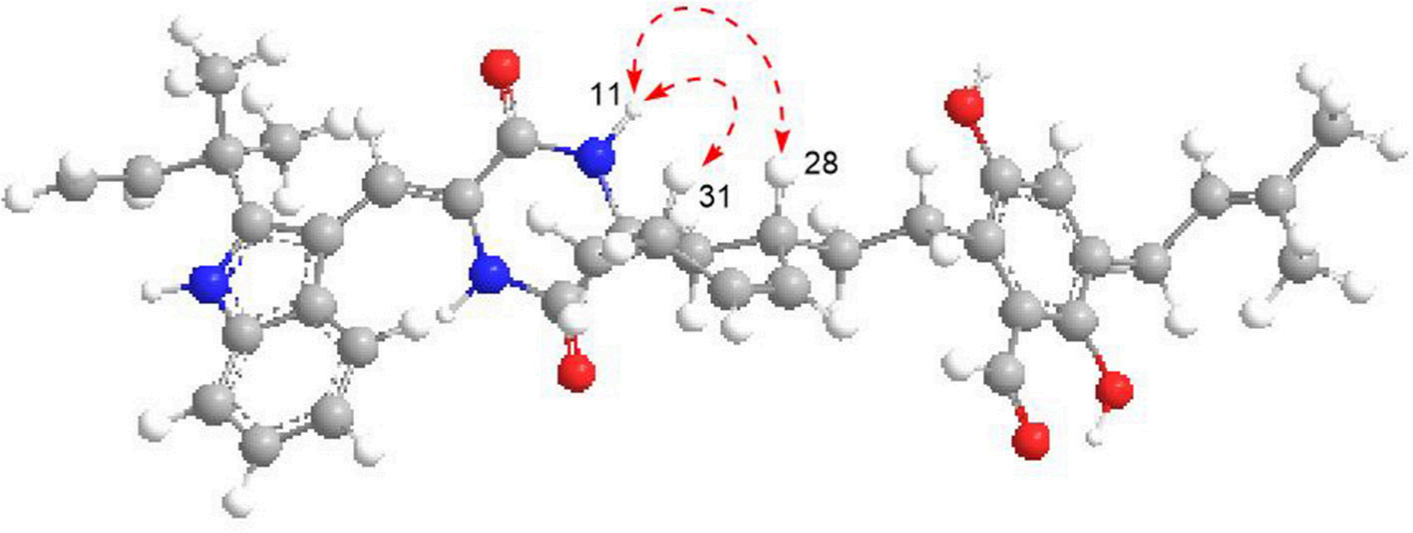
Key NOESY correlations of compound **2**.

Eurotinoid B (**2**) was obtained as a yellow solid. Its molecular formula C_38_H_43_N_3_O_5_ was established by positive HRESIMS at *m/z* 644.3099 [M + Na]^+^ (calcd for 644.3095), which was identical to that of **1**. The UV and 1D NMR spectra (Table 1) of **2** highly resembled those of **1**, except for some small differences of chemical shifts ascribed to the protons and carbons situated in the cyclohexene ring, indicating **2** was a diastereomer of **1**. Further analysis of its ^1^H–^1^H COZY, HSQC, and HMBC NMR data confirmed the above elucidation. The NOE correlations (Figure 4) between H-11 and H-28/H-31 led to their assignment as α-orientation, which implied **2** was a C-28 and C-31 isomer of **1**. Similar to **1**, **2** also occurred as a racemate as justified by the zero specific rotation and baseline ECD curve. Compound **2** was further separated by HPLC using a chiral column to yield a pair of enantiomers (+)-**2** and (–)-**2** (Figure S2). The absolute configurations of (+)-**2** and (–)-**2** were also established by comparison of their experimental ECD spectra with calculated one. Consequently, the calculated ECD for 12*S*,28*S*,31*S*-**2** showed well consistency with the experimental one of (–)-**2** (Figure 3), leading to the unambiguously assignments of 12*R*,28*R*,31*R* for (+)-**2** and 12*S*,28*S*,31*S* for (–)-**2**, respectively.

**Figure 5:**
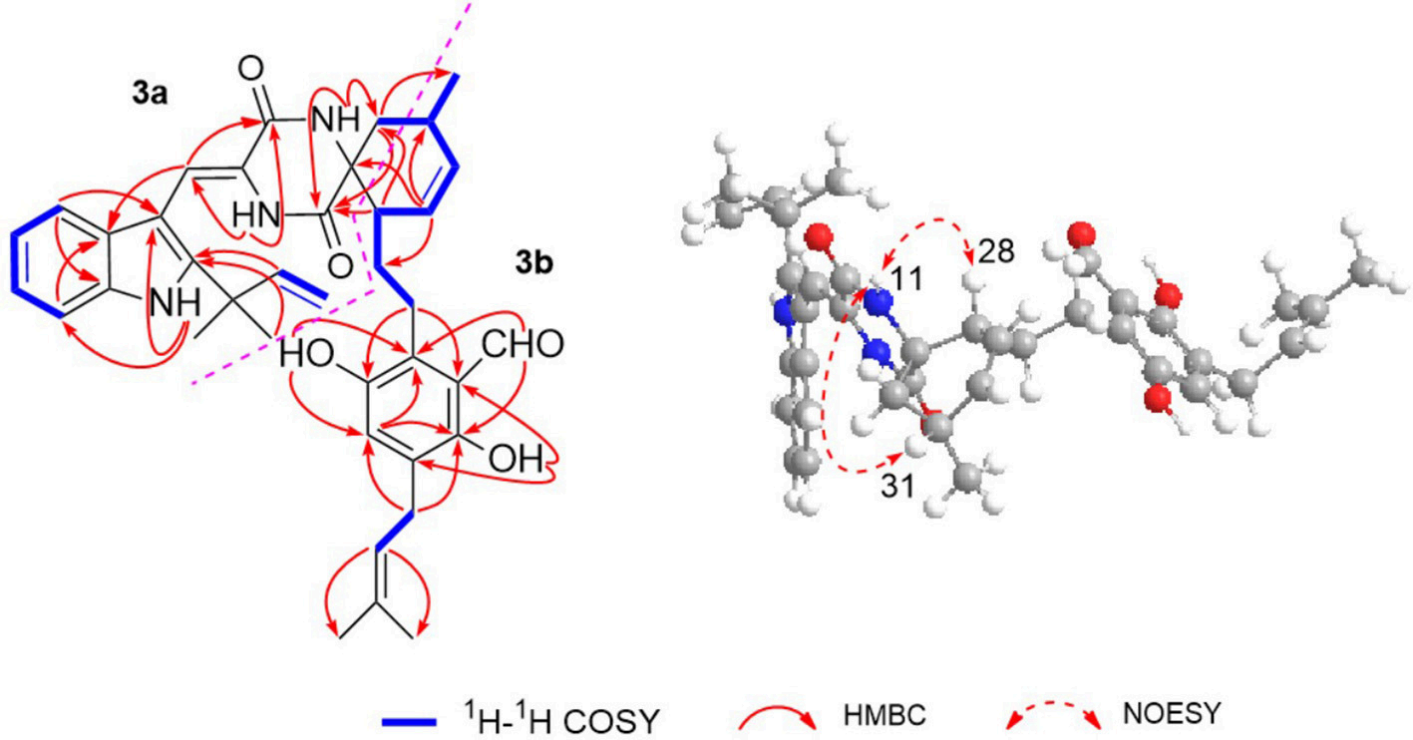
Key ^1^H–^1^H COZY, HMBC, and NOESY correlations of compound **3**.

Eurotinoid C (**3**) was isolated as a yellow solid. Its molecular formula C_38_H_43_N_3_O_5_ was determined on the basis of positive HRESIMS at *m/z* 622.3282 [M + H]^+^ (calcd for 622.3275), corresponding to an index of hydrogen deficiency of 19, which was identical to that of **4**. Its UV and IR spectra displayed typical hydroxyl and amine groups, and carbonyl functionalities. The ^1^H and ^13^C NMR spectra (Table 1) of **3** exhibited characteristic resonances of that of compound **4**. Further analysis of its 2D NMR spectra data allowed us to establish two fragments **3a** and **3b** with their coupling manner similar to **4**, different from **1** and **2**. The key ^1^H–^1^H COZY (Figure 5) correlation between H_2_-32 and H-31, and HMBC correlations between H-11 and C-12/C-13/C-32, H_2_-27 and C-12/C-29, H-28 and C-12/C-13/C-30/C-32, H-29 and C-12/C-31, H_2_-32 and C-12/C-13/C-28/C-30/C-33 clearly supported the above elucidation. Interestingly, the planar structure of **3** was defined to be identical to dihydrocryptoechinulin D (**4**), indicating **3** was a diastereomer of **4**. The above elucidation was verified by the key NOE correlations (Figure 5) between H-11 and H-28/H-31 in **3**, which defined its relative configuration as 12R^*^,28R^*^,31R^*^, revealing **3** was a C-28 and C-31 isomer of **4**. Similarly to **4**, **3** was also a racemic mixture. The absolute configurations of individual (+)-**3** and (–)-**3** were determined by comparison of their experimental and calculated ECD spectra. As a result, the calculated ECD curve for 12*R*,28*R*,31*R*-**3** displayed good agreement with the experimental one of (+)-**3** (Figure S3). Thus, the absolute configurations of (+)-**3** and (–)-**3** were unambiguously assigned as 12*R*,28*R*,31*R* and 12*S*,28*S*,31*S*, respectively.

**Scheme 1:**
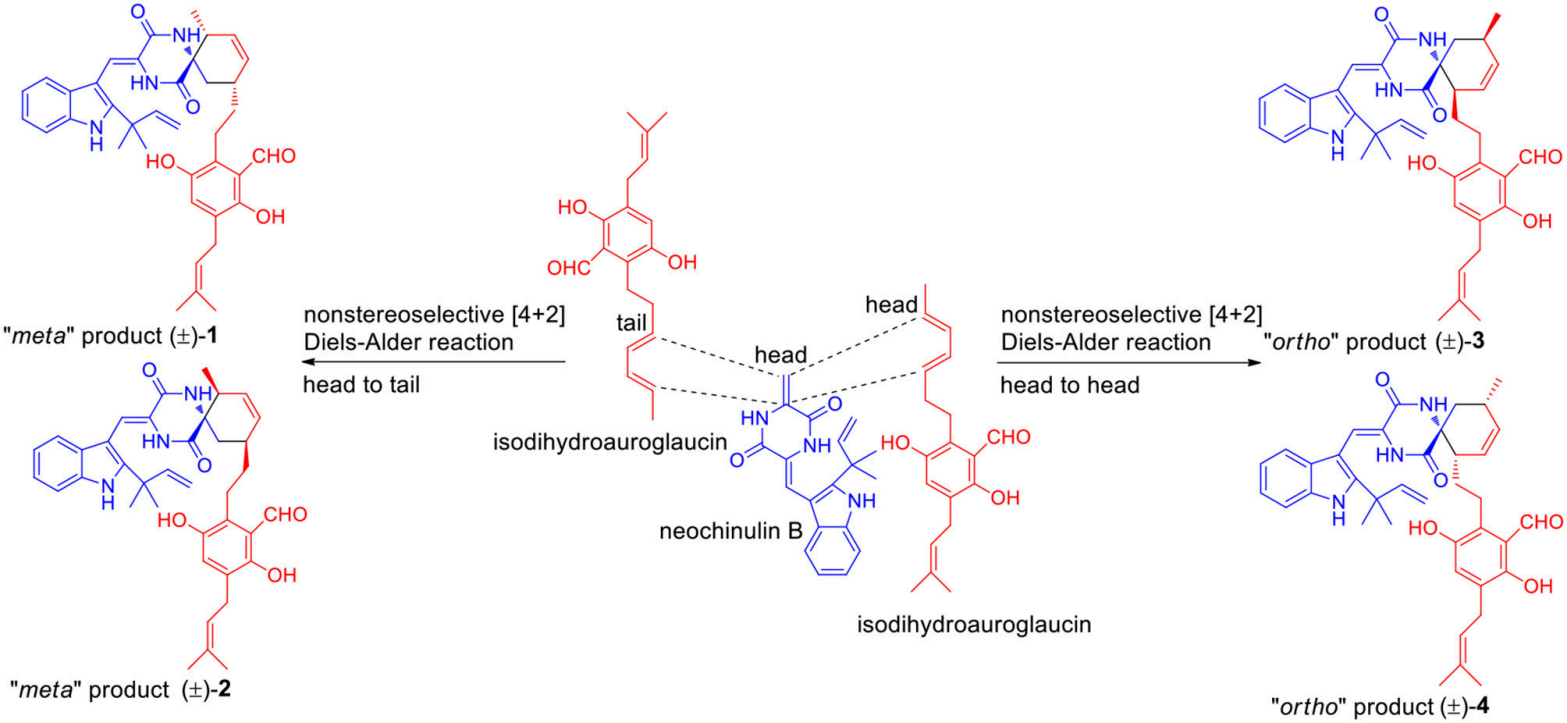
Proposed biosynthetic pathway of compounds **1**–**4**.

The Diels-Alder (DA) reaction is classified as a [4 + 2] cycloaddition in the pericyclic reaction involving a 1,3-diene and a dienophile to afford a six-membered ring with four contiguous stereocenters (Minami and Oikawa, 2016). Hundreds of natural products (NP) containing carbocycles or heterocycles are potentially biosynthesized by DA reaction. With the advancements in whole-genome sequencing and searching tools of biosynthetic gene clusters of secondary metabolites, some Diels-Alderases, such as SpnF, PyrE3, Pyrl4, AbyU, and PvhB, have been identified and substantiated to be of great importance in the biosynthesis of NPs (Takao et al., 2005; Kim et al., 2011; Fage et al., 2015; Tian et al., 2015; Byrne et al., 2016; Tan et al., 2019). Besides, some NPs like spirotriscoumarins A and B (Tang et al., 2016), artemisians A–D (Xue et al., 2017), and alpininoids A–E (Liu et al., 2018) are proposed to be biosynthesized by spontaneous DA cycloaddition. A plausible biogenetic route of **1**–**4** is proposed in Scheme 1. The enone group of neoechinulin B, an indole DKP alkaloid, was supposed to be the dienophile to couple with the diene group of a benzaldehyde derivative, isodihydroauroglaucin, forming the four pairs of enantiomers **1**–**4** through a key non-stereoselective [4 + 2] Diels-Alder cycloaddition. To be specific, the dienophile undergone a DA reaction with the diene by a head-to-tail approach to produce **1** and **2**, and by a head-to-head mode to yield **3** and **4**, respectively. Notably, neoechinulin B and isodihydroauroglaucin both have been isolated from this fungus in previous chemical study (Wang et al., 2013; Zhong W.-M. et al., 2018), which supported the biosynthetic hypothesis. To the best of our knowledge, only four such kind of spirocyclic alkaloids have been reported, namely, cryptoechinuline B (Gatti, 1976), cryptoechinuline D (Gatti, 1976), dihydrocryptoechinulin D (Gao et al., 2012), and effusin A (Gao et al., 2012), all isolated from fungi. Structurally, the former three reported compounds, together with compound **3** were all tend to be “*ortho*” products, while **1** and **2** were represented as the first two “*meta*” products belonging to this kind of spirocyclic alkaloids.

### Biological Evaluation of Compounds (±)-1–**(±)-4**

**Figure 6:**
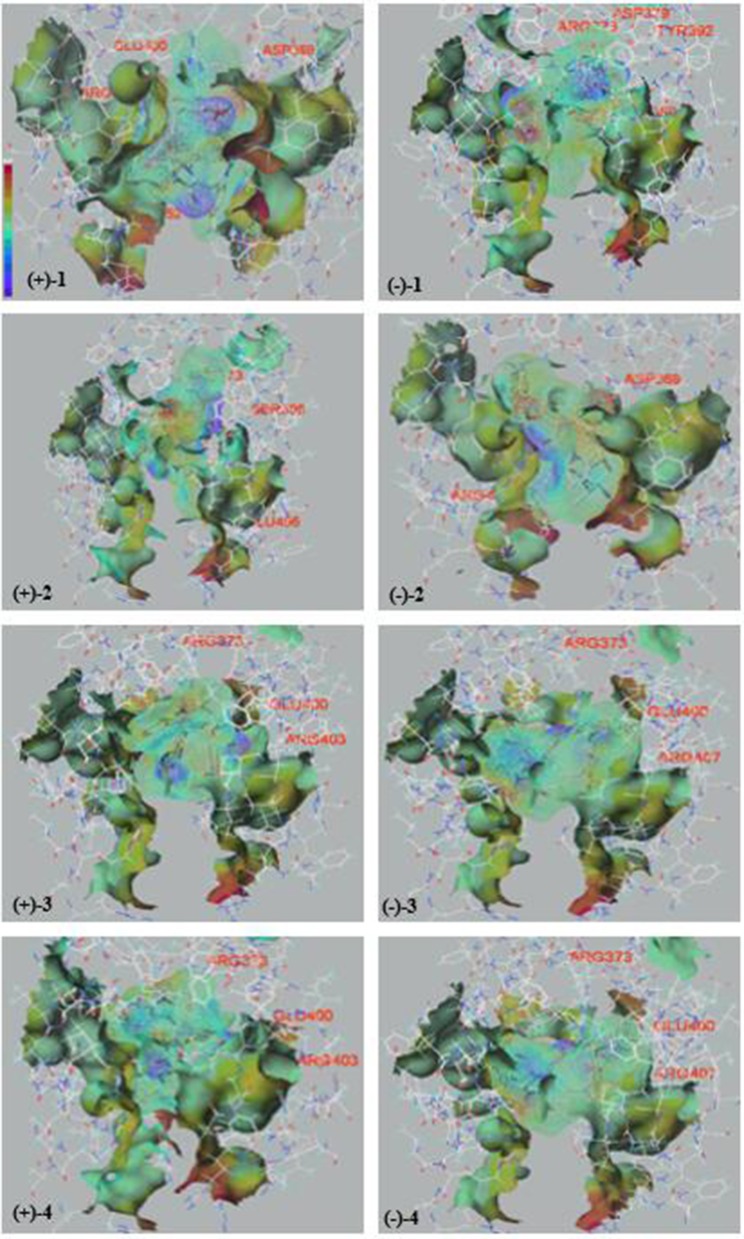
The electrostatic potential and hydrogen-bonds of compounds **(±)-1**–**(±)-4** and the bioactive pockets of 5FNO. (Purple represented stronger electrostatic potential; red represented the residues to form hydrogen-bonds).

We examined (+)-**1**, (–)-**1**, (+)-**2**, (–)-**2**, (+)-**3**, (–)-**3**, (+)-**4**, and (–)-**4** for their antioxidative activities against DPPH and cytotoxic activities against SF-268 and HepG2 cell lines *in vitro* with the SRB method (Zhong W.-M. et al., 2018). All the compounds showed significant radical scavenging activities against DPPH with IC_50_ values ranging from 3.7 to 24.9 μM, which were more potent than that of the positive control ascorbic acid (Vc) (28.4 μM) (Table 2). In addition, compound (+)-**4** showed moderate cytotoxicities against SF-268 and HepG2 cell lines with IC_50_ values of 51.7 ± 2.8, 49.9 ± 2.0 μM, and those of (–)-**4** were 97.3 ± 1.8, 98.7 ± 1.0 μM, respectively (Table 2). Interestingly, according to the bioactivity assay results, (+)-enantiomers exhibited more potent activities than corresponding (–)-enantiomers, indicating that the stereochemistry of the compounds could contribute to their biological activities, which were widely found in the enantiomers of substances, such as *R*-/*S*-thalidomide, *R*-/*S*-warfarin, *R*-/*S*-ehlorpheniramine, and *R*-/*S*-ketamine (Dou et al., 2014). To investigate their bioactive mechanism, 23 and 22 proteins, belonging to five common types of antioxidative targets and six common types of cytotoxic targets respectively, were screened using molecular docking technique. The results (Figure 6 and Figure S7) showed that lipoxygenase (LOX) was the potential antioxidative target for compounds (±)-**1**–(±)-**4**, and epidermal growth factor receptor (EGFR) was the potential cytotoxic target for compounds (±)-**4**, because the trends of detected bioactivities of those compounds and the calculated binding forces between these compounds and proteins of 5FNO (a member of LOX; PDB ID, and so forth) and 4RJ3 (EGFR) were consistent. Furthermore, electrostatic potential contact (EPC) might play critical roles in stabilizing the complexes of the targets and corresponding compounds. Specifically, each isomer of (+)-**1**–(+)-**4** had stronger EPC with the bioactive pocket of 5FNO (a type of LOX; PDB ID, and so forth) than corresponding (–)-**1**–(–)-**4**. Similarly, (+)-**4** possessed tenser EPC with the bioactive pocket of 4RJ3 (EGFR) than (–)-**4**. It revealed proper configurations of the compounds were important for bioactivities.

**Table 2:** Antioxidative activities against DPPH and cytotoxic activities against two tumor cell lines of compounds **(±)-1**–**(±)-4**.

**Compounds**	**IC**_****50****_ **(μM)**		
	**DPPH^**a**^**	**SF-268^**b**^**	**HepG2^**b**^**
(+)-**1**	14.3	>200	>200
(–)-**1**	18.5	>200	>200
(+)-**2**	5.8	>200	>200
(–)-**2**	23.5	>200	>200
(+)-**3**	9.8	>200	>200
(–)-**3**	24.9	>200	>200
(+)-**4**	3.7	51.7 ± 2.8	49.9 ± 2.0
(–)-**4**	6.1	97.3 ± 1.8	98.7 ± 1.0
Vc	23.0		
Taxol		6.0 ± 0.3	11.1 ± 1.1

a *Positive control: Vc*.

b *Positive control: Taxol; The results were mean ±SD (SD = standard deviation)*.

### Physico-Chemical Constants of Compounds 1–3

Eurotinoid A (**1**): yellow solid; [α]${}_{D}^{25}$ = 0 (c 0.05, CH_3_CN); UV (CH_3_CN) λ_max_ (log ε) 203 (4.7), 275 (4.2), 342 (4.2) nm; IR *v*_max_ 3350, 3337, 3314, 2926, 2855, 1682, 1636, 1429, 1207, 1142, 745, 725 cm^−1^; HRESIMS *m/z* 622.3270 [M + H]^+^ (calcd for 622.3275). ^1^H and ^13^C NMR see Table 1.

(+)-**1**: yellow solid; [α]${}_{D}^{25}$ = +200.4 (c 0.05, CH_3_CN); ECD (CH_3_CN) λ_max_ (Δε) 218 (+11.2), 260 (−6.0), 338 (+8.2) nm.

(**–**)-**1**: yellow solid; [α]${}_{D}^{25}$ = −196.8 (c 0.05, CH_3_CN); ECD (CH_3_CN) λ_max_ (Δε) 218 (−8.8), 260 (+5.1), 338 (−7.1) nm.

Eurotinoid B (**2**): yellow solid; [α]${}_{D}^{25}$ = 0 (c 0.05, CH_3_CN); UV (CH_3_CN) λ_max_ (log ε) 225 (5.1), 276 (4.6), 334 (4.5) nm; HRESIMS *m/z* 644.3099 [M + Na]^+^ (calcd for 644.3095). ^1^H and ^13^C NMR see Table 1.

(+)-**2**: yellow solid; [α]${}_{D}^{25}$ = +193.3 (c 0.015, CH_3_CN); ECD (CH_3_CN) λ_max_ (Δε) 209 (+15.9), 260 (−5.9), 328 (+2.9) nm.

(**–**)-**2**: yellow solid; [α]${}_{D}^{25}$ = −200.0 (c 0.015, CH_3_CN); ECD (CH_3_CN) λ_max_ (Δε) 209 (−15.3), 260 (+6.7), 326 (−3.8) nm.

Eurotinoid C (**3**): yellow solid; [α]${}_{D}^{25}$ = 0 (c 0.05, CH_3_CN); UV (CH_3_CN) λ_max_ (log ε) 203 (4.9), 274 (4.4), 347 (4.3) nm; IR *v*_max_ 3364, 2918, 2851, 1715, 1670, 1636, 1541, 1614, 1456, 1412, 1375, 743 cm^−1^; HRESIMS *m/z* 622.3282 [M + H]^+^ (calcd for 622.3275). ^1^H and ^13^C NMR see Table 1.

(+)-**3**: yellow solid; [α]${}_{D}^{25}$ = +212.4 (c 0.025, CH_3_CN); ECD (CH_3_CN) λ_max_ (Δε) 207 (+36.8), 263 (−22.3), 339 (+14.3) nm.

(–)-**3**: yellow solid; [α]${}_{D}^{25}$ = −218.4 (c 0.025, CH_3_CN); ECD (CH_3_CN) λ_max_ (Δε) 207 (−41.9), 262 (+22.2), 339 (−15.1) nm.

## Conclusions

In conclusion, three pairs of new spirocyclic alkaloid enantiomers eurotinoids A–C (**1**–**3**), as well as a known biogenetically related racemate dihydrocryptoechinulin D (**4**), were isolated from a marine-derived fungus *Eurotium* sp. SCSIO F452. Their structures including absolute configurations were determined by extensive spectroscopic data and ECD calculations. Compounds **1** and **2** were represented as the first two “*meta*” products biosynthetically from a non-stereoselective [4 + 2] Diels-Alder cycloaddition presumably between an enone group of a diketopiperazine alkaloid and a diene group of a benzaldehyde derivative via a new head-to-tail coupling mode biosynthetically. All the enantiomers were evaluated antioxidative and cytotoxic activities. A preliminary molecular docking study provided an inside perspective of the action of their different biological activities.

## Author Contributions

WZho performed the isolation, purification, characterization and evaluation the antioxidative activities of all the compounds, and prepared the manuscript. JW contributed to the structure elucidation and revised the manuscript. XW performed the ECD calculations. TF and XH performed the molecular docking experiments. YC and WZha contributed to the determination of cytotoxic activities. QZ and ZH contributed to the isolation of the compounds and determination of antioxidative activities. SZ, LL, and FW designed and supervised the research and revised the manuscript.

### Conflict of Interest Statement

The authors declare that the research was conducted in the absence of any commercial or financial relationships that could be construed as a potential conflict of interest.
