# Supplementary material for: Three Pairs of New Spirocyclic Alkaloid Enantiomers From the Marine-Derived Fungus Eurotium sp. SCSIO F452
Source: Front Chem. 2019 May 20;7:350. doi: 10.3389/fchem.2019.00350 (PMC6536037; doi:10.3389/fchem.2019.00350)
Supplement: Supplementary file 1 [file Data_Sheet_1.pdf]

Three Pairs of New Spirocyclic Alkaloid Enantiomers from the Marine-Derived Fungus *Eurotium* sp. SCSIO F452

Weimao Zhong[1,5], Junfeng Wang[1], Xiaoyi Wei[2], Tingdan Fu[3], Yuchan Chen[4], Qi Zeng[1,5], Zhonghui Huang[1,5], Xinan Huang[3], Weimin Zhang[4], Si Zhang[1], Lijuan Long[1]* and Fazuo Wang[1]*

[1] CAS Key Laboratory of Tropical Marine Bio-resources and Ecology, Guangdong Key Laboratory of Marine Materia Medica, RNAM Center for Marine Microbiology, South China Sea Institute of Oceanology, Chinese Academy of Sciences, 164 West Xingang Road, Guangzhou 510301, China, [2] Key Laboratory of Plant Resources Conservation and Sustainable Utilization, South China Botanical Garden, Chinese Academy of Sciences, Guangzhou 510650, China, [3] Institute of Tropical Medicine, Guangzhou University of Chinese Medicine, Guangzhou 510400, China, [4] State Key Laboratory of Applied Microbiology Southern China, Guangdong Provincial Key Laboratory of Microbial Culture Collection and Application, Guangdong Open Laboratory of Applied Microbiology, Guangdong Institute of Microbiology, 100 Central Xianlie Road, Guangzhou 510070, China, [5] University of Chinese Academy of Sciences, 19 Yuquan Road, Beijing 100049, China

***Correspondence:**

Lijuan Long

longlj@scsio.ac.cn

Fazuo Wang

wangfazuo@scsio.ac.cn

Table of Contents

Experimental Details 3

Computational details 4

Molecular docking study 6

References 13

Figure S8 The ^{1}H NMR spectrum of eurotinoid A (**1**) in CD_3COCD_3. 15

Figure S9 The ^{13}C NMR spectrum of eurotinoid A (**1**) in CD_3COCD_3. 16

Figure S10 The HSQC spectrum of eurotinoid A (**1**) in CD_3COCD_3. 17

Figure S11 The HMBC spectrum of eurotinoid A (**1**) in CD_3COCD_3. 18

Figure S12 The ^{1}H–^{1}H COSY spectrum of eurotinoid A (**1**) in CD_3COCD_3. 19

Figure S13 The NOESY spectrum of eurotinoid A (**1**) in CD_3COCD_3. 20

Figure S14 The HRESIMS spectrum of eurotinoid A (**1**) in CD_3COCD_3. 21

Figure S15 The IR spectrum of eurotinoid A (**1**) in CD_3COCD_3. 22

Figure S16 The UV spectrum of eurotinoid A (**1**) in CD_3COCD_3. 23

Figure S17 The ^{1}H NMR spectrum of eurotinoid B (**2**) in CD_3COCD_3. 24

Figure S18 The ^{13}C NMR spectrum of eurotinoid B (**2**) in CD_3COCD_3. 25

Figure S19 The HSQC spectrum of eurotinoid B (**2**) in CD_3COCD_3. 26

Figure S20 The HMBC spectrum of eurotinoid B (**2**) in CD_3COCD_3. 27

Figure S21 The ^{1}H–^{1}H COSY spectrum of eurotinoid B (**2**) in CD_3COCD_3. 28

Figure S22 The NOESY spectrum of eurotinoid B (**2**) in CD_3COCD_3. 29

Figure S23 The HRESIMS spectrum of eurotinoid B (**2**). 30

Figure S24 The UV spectrum of eurotinoid B (**2**). 31

Figure S25 The ^{1}H NMR spectrum of eurotinoid C (**3**) in DMSO-d_6. 32

Figure S26 The ^{13}C NMR spectrum of eurotinoid C (**3**) in DMSO-d_6. 33

Figure S27 The HSQC spectrum of eurotinoid C (**3**) in DMSO-d_6. 34

Figure S28 The HMBC spectrum of eurotinoid C (**3**) in DMSO-d_6. 35

Figure S29 The ^{1}H–^{1}H COSY spectrum of eurotinoid C (**3**) in DMSO-d_6. 36

Figure S30 The NOESY spectrum of eurotinoid C (**3**) in DMSO-d_6. 37

Figure S31 The HRESIMS spectrum of eurotinoid C (**3**). 38

Figure S32 The IR spectrum of eurotinoid C (**3**). 39

Figure S33 The UV spectrum of eurotinoid C (**3**). 40

Experimental Details

Chiral separation

The chiral HPLC separation of 1.

Chromatographic conditions:

(1) Column: Daicel chiralpak IC (250 × 4.6 mm, 5 μm)

(2) Mobile phase: n-hexane/isopropanol (87:13)

(3) Wavelength: 210 nm, 254 nm, 280 nm

(4) Flow rate: 1 mL/min

(5) Retention time: (+)-**1** (10.825 min), (–)-**1** (23.026 min)

(6) Yield: (+)-**1** (1.3 mg), (–)-**1** (1.4 mg)

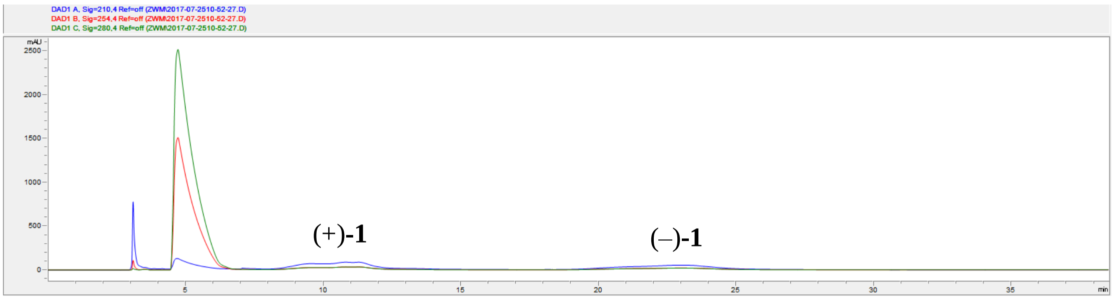

Figure S1 The chiral HPLC chromatogram of **1**.

The chiral HPLC separation of 2.

Chromatographic conditions:

(1) Column: Daicel chiralpak IA (250 × 4.6 mm, 5 μm)

(2) Mobile phase: n-hexane/isopropanol (72:28)

(3) Wavelength: 202 nm, 254 nm, 280 nm

(4) Flow rate: 0.9 mL/min

(5) Retention time: (+)-**2** (6.379 min), (–)-**2** (7.053 min)

(6) Yield: (+)-**2** (0.6 mg), (–)-**2** (0.6 mg)

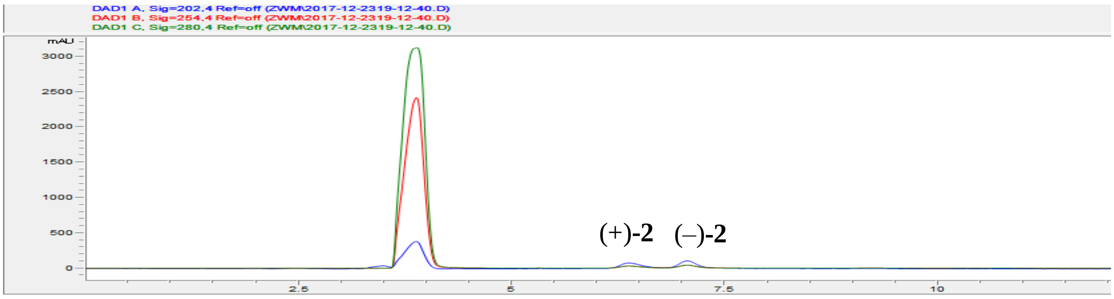

Figure S2 The chiral HPLC chromatogram of **2**.

The chiral HPLC separation of 3.

(1) Column: Daicel chiralpak IC (250 × 4.6 mm, 5 μm)

(2) Mobile phase: n-hexane/isopropanol (87:13)

(3) Wavelength: 210 nm, 254 nm, 280 nm

(4) Flow rate: 1 mL/min

(5) Retention time: (+)-**3** (11.887 min), (–)-**3** (17.220 min)

(6) Yield: (+)-**3** (0.8 mg), (–)-**3** (0.8 mg)

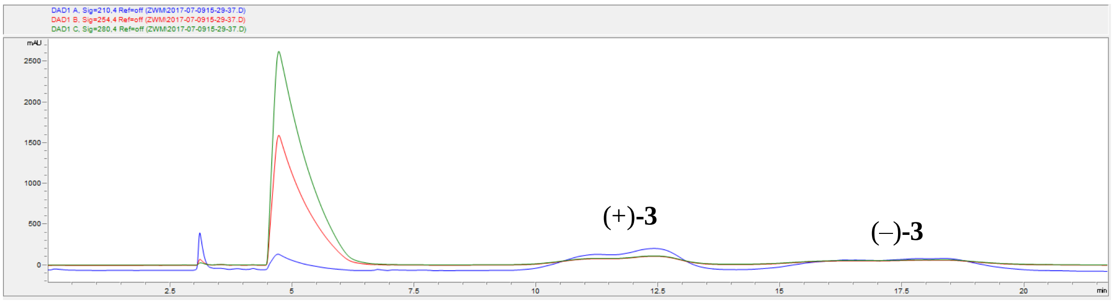

Figure S3 The chiral HPLC chromatogram of **3**.

The chiral HPLC separation of 4.

(1) Column: Daicel chiralpak IC (250 × 4.6 mm, 5 μm)

(2) Mobile phase: n-hexane/isopropanol (90:10)

(3) Wavelength: 210 nm, 254 nm, 280 nm

(4) Flow rate: 1 mL/min

(5) Retention time: (+)-**4** (22.237 min), (–)-**4** (34.315 min)

(6) Yield: (+)-**4** (1.8 mg), (–)-**4** (1.7 mg)

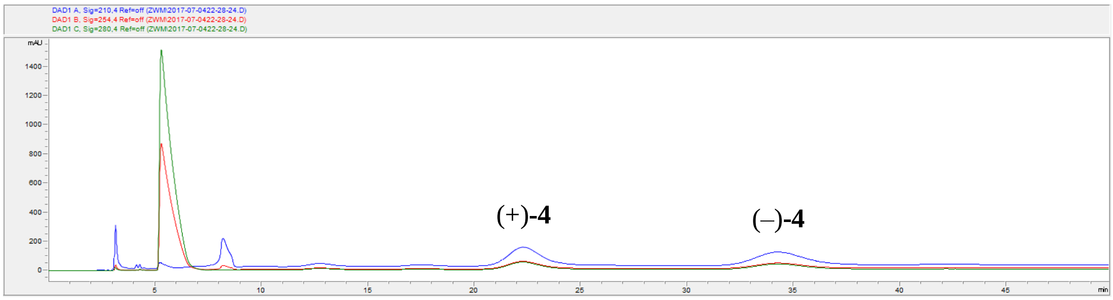

Figure S4 The chiral HPLC chromatogram of **4**.

Computational details

1. Methods

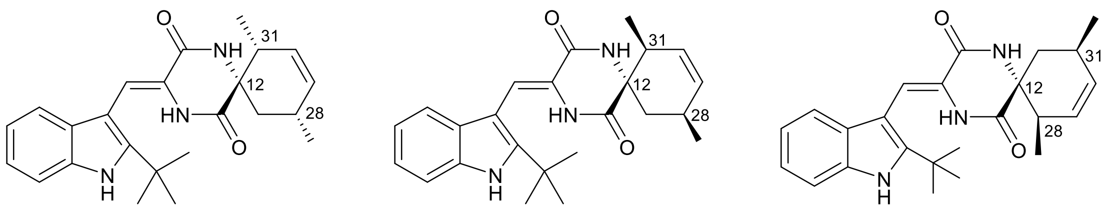

1' **2'** **3'**

Figure S5 Structures of the truncated model compounds of (12*S*,28*R*,31*R*)-**1'**, (12*S*,28*S*,31*S*)-**2'**, and (12*R*,28*R*,31*R*)-**3'** applied for theoretical calculations.

2. Results

Table S1 Relative thermal energies (ΔE), relative free energies (ΔG), and equilibrium populations (P) of low-energy conformers of structures (12*S*,28*R*,31*R*)-**1'**, (12*S*,28*S*,31*S*)-**2'**, and (12*R*,28*R*,31*R*)-**3'** in MeCN.

Conformer	Δ*E* (kcal/mol) [a]	Δ*G* (kcal/mol) [a]	P (%)[b]
Compound (12*S*,28*R*,31*R*)-**1'**			
**1'a**	0.0	0.0	79.3
**1'b**	1.21	0.83	19.7
**1'c** [c]	2.77	2.59	1.0
Compound (12*S*,28*S*,31*S*)-**2'**			
**2'a**	0.0	0.0	80.3
**2'b**	1.08	0.84	19.4
**2'c** [c]	2.81	3.41	0.3
Compound (12*R*,28*R*,31*R*)-**3'**			
**3'a**	0.0	0.0	89.8
**3'b**	1.52	1.30	10.1
**3'c** [c]	3.63	4.06	0.1

[a] At the M06-2X/def2-TZVP/ IEFPCM level of theory.

[b] From Δ*G* values at 298.15 K.

[c] Conformer not applied to ECD/TDDFT calculations.

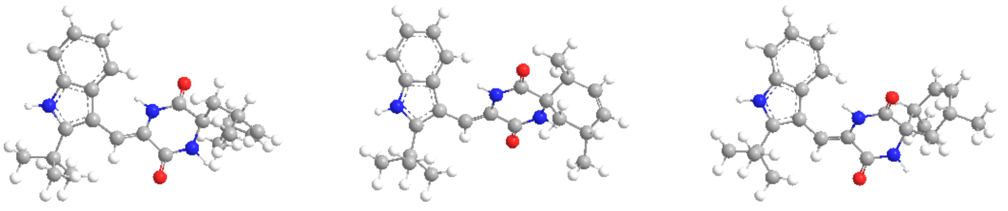

1'a **1'b** **1'c**

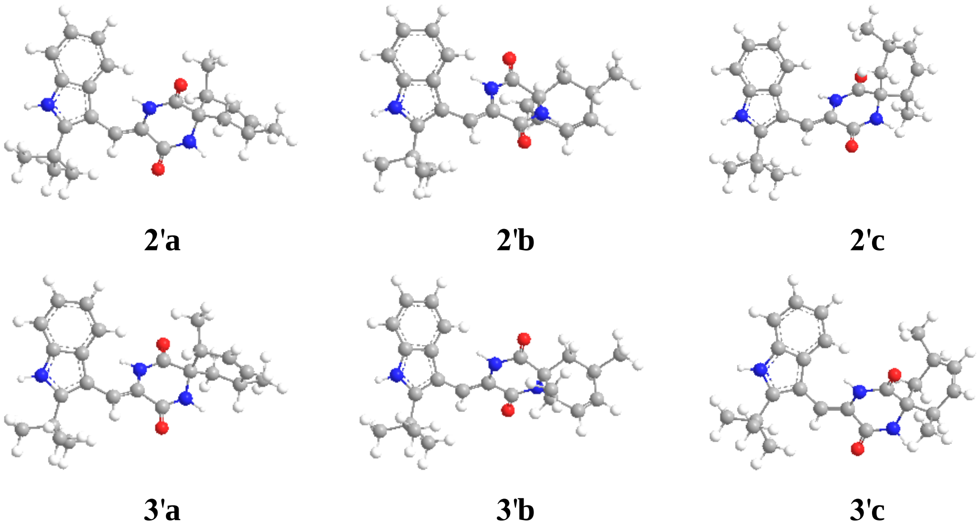

Figure S6 Conformations of low-energy conformers of (12*S*,28*R*,31*R*)-**1**', (12*S*,28*S*,31*S*)-**2**', and (12*R*,28*R*,31*R*)-**3**'.

Molecular docking study

1. Methods

According to the references, five common types of antioxidative targets, such as lipoxygenase (LOX) (Mashima and Okuyama 2015), superoxide dismutase (SOD) (Tovmasyan et al., 2014), glutathione peroxidase (GSH-PX) (Jin et al., 2015), xanthine oxidase (XOD) (Kelley et al., 2010), and peroxiredoxin (PRDX) (Chae et al., 2017), and six types of cytotoxic targets of epidermal growth factor receptor (EGFR) (Ohashi et al., 2018), vascular endothelial growth factor receptor (VEGFR) (Graziani et al., 2016), cyclin dependent kinases (CDK) (Premnath et al., 2015), focal adhesion kinase (FAK) (Liu et al., 2010), farnesyltransferase (FTase) (Sulzmaier et al., 2014), and B cell lymphoma/leukaemia 2 (Bcl-2) (Bate-Eya et al., 2016) were chosen for bioactive screening of these compounds. The 3D structural data of the proteins in these eleven types of proteins were downloaded and corrected by Sybyl-X 2.1 software package, respectively. After the diverse conformers of each compound were generated, the binding affinity of compound (+)-**4** with antioxidative proteins (Table S2) and cytotoxic proteins (Table S3) were calculated via molecular docking using Surflex-Dock, respectively. The total score of greater than 7 was used as the criterion for filtering the bioactive protein. Then the total score and consensus score (CScore) were analyzed for evaluating the binding affinity.

2. Results

With the total score greater than 7, 5FNO, 1LOX, 1N8Q, 1YGE, 3BNB, 1JNQ, 3PZW, 1ROV, 3BNC, 1B06, 3SOP, 1GP1, 1YZX, 2RM5, 2P31, 1VLB, 3L8W, 4XCS, 3HY2, 2RII, 3TJG and 2WFC (Table S4), as well as 1M17, 4RJ3,1M6B, 1RV6, 3HNG, 5EX3, 1Y6B, 4BSK, 3DDQ, 1PW2, 2R3G, 1DKS, 5TO8, 2AEH, 1OW8, 2R2L, 4GTM, 1D8E, and 2YXJ (Table S5) were selected as the candidates for the next round of virtual screening. These eight compounds were classified into four pairs of isomers, and in each pair the compound with (+)-configuration exhibited higher bioactivity in bioassay. On the comprehensive consideration of the bioactivity, total score and Cscore (Tables S4 and S5), 5FNO and 4RJ3 were suggeted to be potential targets for antioxidation and cytotoxicity, respectively. The contribution of force fields for each binding between the protein and crossponding compound were listed in Table S6, which demonstrated the ChemScores of Cscore were relatively consistent with the binding affinity in each pair of isomers. It suggeted that the electrostatic potential contact may play critical roles in the binding (Figures. 6 and S7).

Table S2 antioxidative proteins

Targets	PDB ID
LOX	5FNO, 1LOX, 1N8Q, 1YGE, 3BNB, 3PZW, 1ROV, 3BNC
SOD	1B06, 3SOP
GSH-PX	1GP1, 1YZX, 2RM5, 2P31,4EVM
XOD	1VLB, 3L8W
PRDX	4XCS, 3HY2, 2RII, 3TJG, 2WFC, 4K7N

Table S3 cytotoxicity proteins

Targets	PDB ID
EGFR	1M17, 3W2S, 4RJ3, 1M6B
VEGFR	1RV6, 3HNG, 5EX3, 1Y6B, 4BSK
CDK	3DDQ, 1PW2, 2R3G, 1DKS
FAK	5TO8, 2AEH, 1KTM, 1OW8, 4XEF
FTase	2R2L, 4GTM, 1D8E
Bcl-2	2YXJ

Table S4 Molecular docking for compounds (±)-**1**–(±)-**4**.

PDB ID	Chemical	Total Score	CSCORE
	(+)-**1**	7.383	3

5FNO	(–)-**1**	7.214	0
	(+)-**2**	13.151	4
	(–)-**2**	12.433	4
	(+)-**3**	14.428	4
	(–)-**3**	13.050	3
	(+)-**4**	14.428	4
	(–)-**4**	13.050	3
1LOX	(+)-**1**	12.201	4
	(–)-**1**	12.422	3
	(+)-**2**	12.577	2
	(–)-**2**	15.969	3
	(+)-**3**	13.272	3
	(–)-**3**	13.377	2
	(+)-**4**	13.272	3
	(–)-**4**	13.377	2
1N8Q	(+)-**1**	13.603	1
	(–)-**1**	14.095	0
	(+)-**2**	14.555	1
	(–)-**2**	16.405	2
	(+)-**3**	15.584	3
	(–)-**3**	12.588	3
	(+)-**4**	15.584	3
	(–)-**4**	12.588	3
1YGE	(+)-**1**	15.069	2
	(–)-**1**	13.271	2
	(+)-**2**	13.300	0
	(–)-**2**	13.300	2
	(+)-**3**	14.441	3
	(–)-**3**	16.281	1
	(+)-**4**	14.441	3
	(–)-**4**	16.281	1
3BNB	(+)-**1**	12.865	0
	(–)-**1**	13.757	0
	(+)-**2**	14.960	1
	(–)-**2**	14.182	2
	(+)-**3**	14.472	1
	(–)-**3**	11.416	2
	(+)-**4**	14.472	1
	(–)-**4**	11.416	2
1JNQ	(+)-**1**	11.019	0
	(–)-**1**	11.851	0
	(+)-**2**	9.470	0
	(–)-**2**	6.815	0
	(+)-**3**	12.180	4

	(–)-**3**	12.573	1
	(+)-**4**	12.180	4
	(–)-**4**	12.573	1
3PZW	(+)-**1**	12.962	2
	(–)-**1**	12.973	1
	(+)-**2**	16.253	4
	(–)-**2**	13.583	0
	(+)-**3**	11.890	2
	(–)-**3**	12.192	2
	(+)-**4**	11.890	2
	(–)-**4**	12.192	2
1ROV	(+)-**1**	13.121	1
	(–)-**1**	10.215	1
	(+)-**2**	10.661	2
	(–)-**2**	9.422	3
	(+)-**3**	10.659	2
	(–)-**3**	10.668	4
	(+)-**4**	10.659	2
	(–)-**4**	10.668	4
3BNC	(+)-**1**	12.404	1
	(–)-**1**	13.602	0
	(+)-**2**	12.216	5
	(–)-**2**	12.894	4
	(+)-**3**	14.957	2
	(–)-**3**	12.822	4
	(+)-**4**	14.957	2
	(–)-**4**	12.822	4
1B06	(+)-**1**	9.811	1
	(–)-**1**	9.954	1
	(+)-**2**	10.100	4
	(–)-**2**	10.430	3
	(+)-**3**	7.812	5
	(–)-**3**	8.050	3
	(+)-**4**	7.812	5
	(–)-**4**	8.050	3
3SOP	(+)-**1**	12.590	0
	(–)-**1**	10.970	2
	(+)-**2**	10.102	3
	(–)-**2**	10.526	4
	(+)-**3**	9.801	2
	(–)-**3**	12.486	3
	(+)-**4**	9.801	2
	(–)-**4**	12.486	3
	(+)-**1**	9.550	1

1GP1	(–)-**1**	8.704	0
	(+)-**2**	8.851	1
	(–)-**2**	9.163	2
	(+)-**3**	10.250	1
	(–)-**3**	8.614	4
	(+)-**4**	10.250	1
	(–)-**4**	8.614	4
1YZX	(+)-**1**	14.268	4
	(–)-**1**	14.692	1
	(+)-**2**	11.905	4
	(–)-**2**	10.851	1
	(+)-**3**	11.013	4
	(–)-**3**	14.330	2
	(+)-**4**	11.013	4
	(–)-**4**	14.330	2
2RM5	(+)-**1**	6.891	1
	(–)-**1**	7.735	1
	(+)-**2**	7.767	1
	(–)-**2**	6.937	1
	(+)-**3**	7.137	4
	(–)-**3**	9.026	0
	(+)-**4**	7.137	4
	(–)-**4**	9.026	0
2P31	(+)-**1**	4.168	0
	(–)-**1**	9.354	1
	(+)-**2**	13.152	0
	(–)-**2**	6.292	0
	(+)-**3**	8.270	5
	(–)-**3**	6.095	1
	(+)-**4**	8.270	5
	(–)-**4**	6.095	1
1VLB	(+)-**1**	10.052	4
	(–)-**1**	9.976	2
	(+)-**2**	11.706	2
	(–)-**2**	11.924	2
	(+)-**3**	12.257	2
	(–)-**3**	10.918	2
	(+)-**4**	12.257	2
	(–)-**4**	10.918	2
3L8W	(+)-**1**	8.056	2
	(–)-**1**	10.086	1
	(+)-**2**	8.853	1
	(–)-**2**	7.413	1
	(+)-**3**	9.670	4

	(–)-**3**	7.898	1
	(+)-**4**	9.670	4
	(–)-**4**	7.898	1
4XCS	(+)-**1**	10.180	3
	(–)-**1**	13.691	0
	(+)-**2**	11.074	2
	(–)-**2**	14.762	2
	(+)-**3**	11.128	1
	(–)-**3**	16.507	1
	(+)-**4**	11.128	1
	(–)-**4**	16.507	1
3HY2	(+)-**1**	11.254	1
	(–)-**1**	11.365	1
	(+)-**2**	9.097	0
	(–)-**2**	12.783	4
	(+)-**3**	10.893	4
	(–)-**3**	11.909	0
	(+)-**4**	10.893	4
	(–)-**4**	11.909	0
2RII	(+)-**1**	10.281	1
	(–)-**1**	10.980	3
	(+)-**2**	13.143	2
	(–)-**2**	10.731	4
	(+)-**3**	12.723	0
	(–)-**3**	14.650	1
	(+)-**4**	12.722	0
	(–)-**4**	14.650	1
3TJG	(+)-**1**	11.169	0
	(–)-**1**	11.801	1
	(+)-**2**	11.336	2
	(–)-**2**	14.325	4
	(+)-**3**	9.360	1
	(–)-**3**	11.528	0
	(+)-**4**	9.360	1
	(–)-**4**	11.528	0
2WFC	(+)-**1**	14.008	0
	(–)-**1**	13.517	0
	(+)-**2**	11.086	0
	(–)-**2**	12.161	3
	(+)-**3**	14.456	0
	(–)-**3**	11.215	1
	(+)-**4**	14.456	0
	(–)-**4**	11.215	1
4EVM	(+)-**4**	6.376	0

4K7N	(+)-**4**	6.001	2

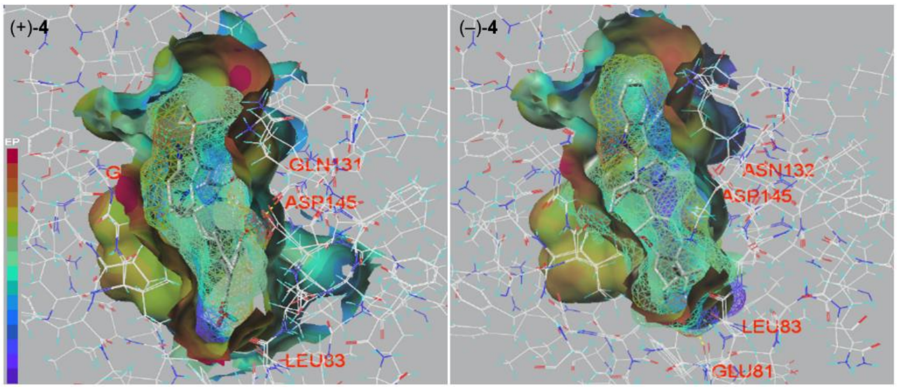

Figure S7 The electrostatic potential and hydrogen-bonds of compounds (+)-**4**/(−)-**4** and the bioactive pocket of 4RJ3. (Purple represented stronger electrostatic potential; red represented the residues to form hydrogen-bonds.)

Table S5 Molecular docking for compounds (±)-**4**.

PDB ID	Chemical	Total Score	CSCORE
1M17	(+)-**4**	8.866	4
	(+)-**4**	10.926	2
4RJ3	(+)-**4**	10.812	4
	(+)-**4**	7.964	1
1M6B	(+)-**4**	12.365	0
	(+)-**4**	9.577	2
1RV6	(+)-**4**	7.181	0
	(+)-**4**	11.077	0
3HNG	(+)-**4**	12.726	2
	(+)-**4**	11.952	4
5EX3	(+)-**4**	11.743	3
	(+)-**4**	13.817	2
1Y6B	(+)-**4**	7.049	4
	(+)-**4**	8.655	1
4BSK	(+)-**4**	8.294	5
	(+)-**4**	2.263	3
3DDQ	(+)-**4**	10.632	4
	(+)-**4**	12.735	2
1PW2	(+)-**4**	10.474	0
	(+)-**4**	15.254	1
2R3G	(+)-**4**	11.812	4
	(+)-**4**	14.047	5
1DKS	(+)-**4**	7.156	1
	(+)-**4**	8.774	4
5TO8	(+)-**4**	8.503	5
	(+)-**4**	9.048	5
2AEH	(+)-**4**	8.490	4

	(+)-**4**	10.353	2
1OW8	(+)-**4**	9.378	5
	(+)-**4**	9.405	1
2R2L	(+)-**4**	10.309	4
	(+)-**4**	11.382	4
4GTM	(+)-**4**	9.370	1
	(+)-**4**	10.607	0
1D8E	(+)-**4**	11.427	4
	(+)-**4**	12.144	0
2YXJ	(+)-**4**	8.475	1
	(+)-**4**	5.757	1
1KTM	(+)-**4**	6.165	4
4XEF	(+)-**4**	5.929	3

Table S6 Molecular docking for compounds (±)-**1**–(±)-**4**.

PDB ID	Chemical	Total Score	CScore	ChemScore	G-Score	D-Score	PMF-Score
5FNO	(+)-**1**	7.384	3	-27.041	-319.896	-152.844	-142.035
	(–)-**1**	7.214	0	-20.820	-279.620	-149.315	-125.443
	(+)-**2**	13.151	4	-34.042	-324.907	-186.325	-170.406
	(–)-**2**	12.433	4	-28.527	-330.850	-165.110	-136.211
	(+)-**3**	14.428	4	-29.916	-304.492	-161.329	-134.328
	(–)-**3**	13.050	3	-27.888	-324.571	-167.781	-116.948
	(+)-**4**	14.428	4	-29.916	-304.492	-161.329	-134.328
	(–)-**4**	13.050	3	-27.888	-324.571	-167.781	-116.948
4RJ3	(+)-**4**	10.812	4	-39.821	-388.211	-209.607	-15.828
	(–)-**4**	7.964	1	-34.404	-330.191	-190.883	-27.673

References

Tovmasyan, A., Reboucas, J. S., and Benov, L. (2014). Simple biological systems for assessing the activity of superoxide dismutase mimics. *Antioxid. Redox. Signal.* 20, 2416-2436. doi: 10.1089/ars.2013.5576

Bate-Eya, L. T., Den, H. I. J. M., Ida, V. D. P., Linda, S., Jan, K., Santo, E. E., et al. (2016). High efficacy of the BCL-2 inhibitor ABT199 (venetoclax) in BCL-2 high-expressing neuroblastoma cell lines and xenografts and rational for combination with MCL-1 inhibition. *Oncotarget*. 7, 27946-27958. doi: 10.18632/oncotarget.8547

Chae, S., Lee, H. K., Kim, Y. K., Jung Sim, H., Ji, Y., Kim, C., et al. (2017). Peroxiredoxin 1, a novel regulator of pronephros development, influences retinoic acid and Wnt signaling by controlling ROS levels. *Sci. Rep*. 7, 8874. doi: 10.1038/s41598-017-09262-6

Graziani, G., Ruffini, F., Tentori, L., Scimeca, M., Dorio, A. S., Atzori, M. G., et al. (2016). Antitumor activity of a novel anti-vascular endothelial growth factor receptor-1 monoclonal antibody that does not interfere with ligand binding. *Oncotarget*. 7, 72868-72885. doi: 10.18632/oncotarget.12108

Jin, L., Li, D., Alesi Gina, N., Fan, J., Kang, H. B., et al. (2015). Glutamate dehydrogenase 1 signals through antioxidant glutathione peroxidase 1 to regulate redox homeostasis and tumor growth.

Cancer Cell. 27, 257-270. doi: 10.1016/j.ccell.2014.12.006

Kelley, E. E., Khoo, N. K., Hundley, N. J., Malik, U. Z., Freeman, B. A., and Tarpey, M. M. (2010). Hydrogen peroxide is the major oxidant product of xanthine oxidase. *Free. Radic. Biol. Med.* 48, 493-498. doi: 10.1016/j.freeradbiomed.2009.11.012

Liu, M., Sjogren, A. K., Karlsson, C., Ibrahim, M. X., Andersson, K. M., Olofsson, F. J., et al. (2010). Targeting the protein prenyltransferases efficiently reduces tumor development in mice with K-RAS-induced lung cancer. *Proc. Natl. Acad. Sci. USA* 107, 6471-6476. doi: 10.1073/pnas.0908396107

Mashima, R., and Okuyama, T. (2015). The role of lipoxygenases in pathophysiology; new insights and future perspectives. *Redox. Biol*. 6, 297-310. doi: 10.1016/j.redox.2015.08.006

Ohashi, Y., Okamura, M., Katayama, R., Fang, S., Tsutsui, S., Akatsuka, A., et al. (2018). Targeting the Golgi apparatus to overcome acquired resistance of non-small cell lung cancer cells to EGFR tyrosine kinase inhibitors. *Oncotarget*. 9, 1641-1655. doi: 10.18632/oncotarget.22895

Premnath, P. N., Craig, S. N., Liu, S., Anderson, E. L., Grigoroudis, A. I., Kontopidis, G., et al. (2015). Iterative conversion of cyclin binding groove peptides into druglike CDK inhibitors with antitumor activity. *J. Med. Chem.* 58, 433-442. doi: 10.1021/jm5015023

Sulzmaier, F. J., Jean, C., and Schlaepfer, D. D. (2015). FAK in cancer: mechanistic findings and clinical applications. *Nat. Rev. Cancer*. 14, 598-610. doi: 10.1038/nrc3792

Figure S8 The ^{1}H NMR spectrum of eurotinoid A (**1**) in CD_3COCD_3.

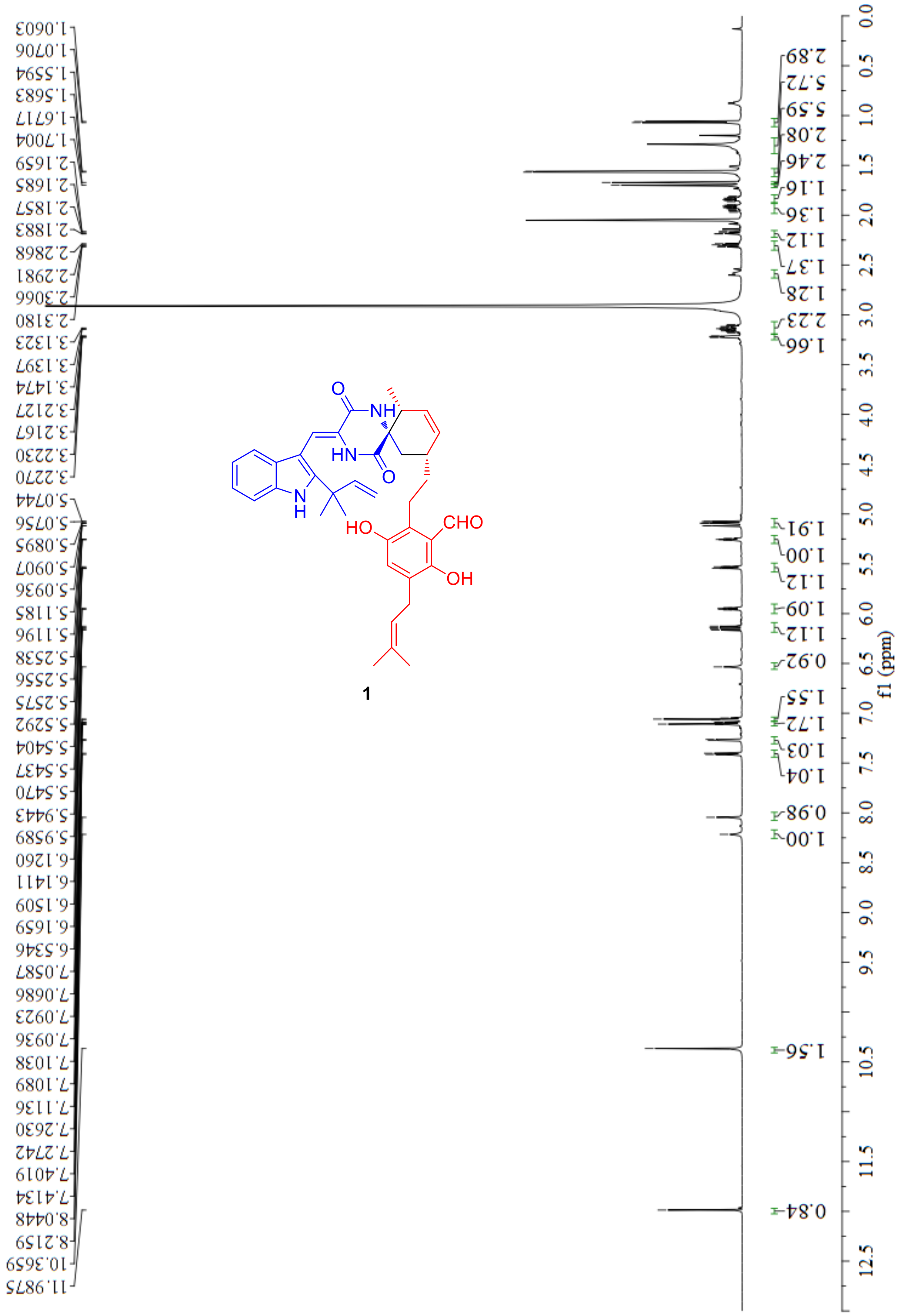

Figure S9 The ^{13}C NMR spectrum of eurotinoid A (**1**) in CD_3COCD_3.

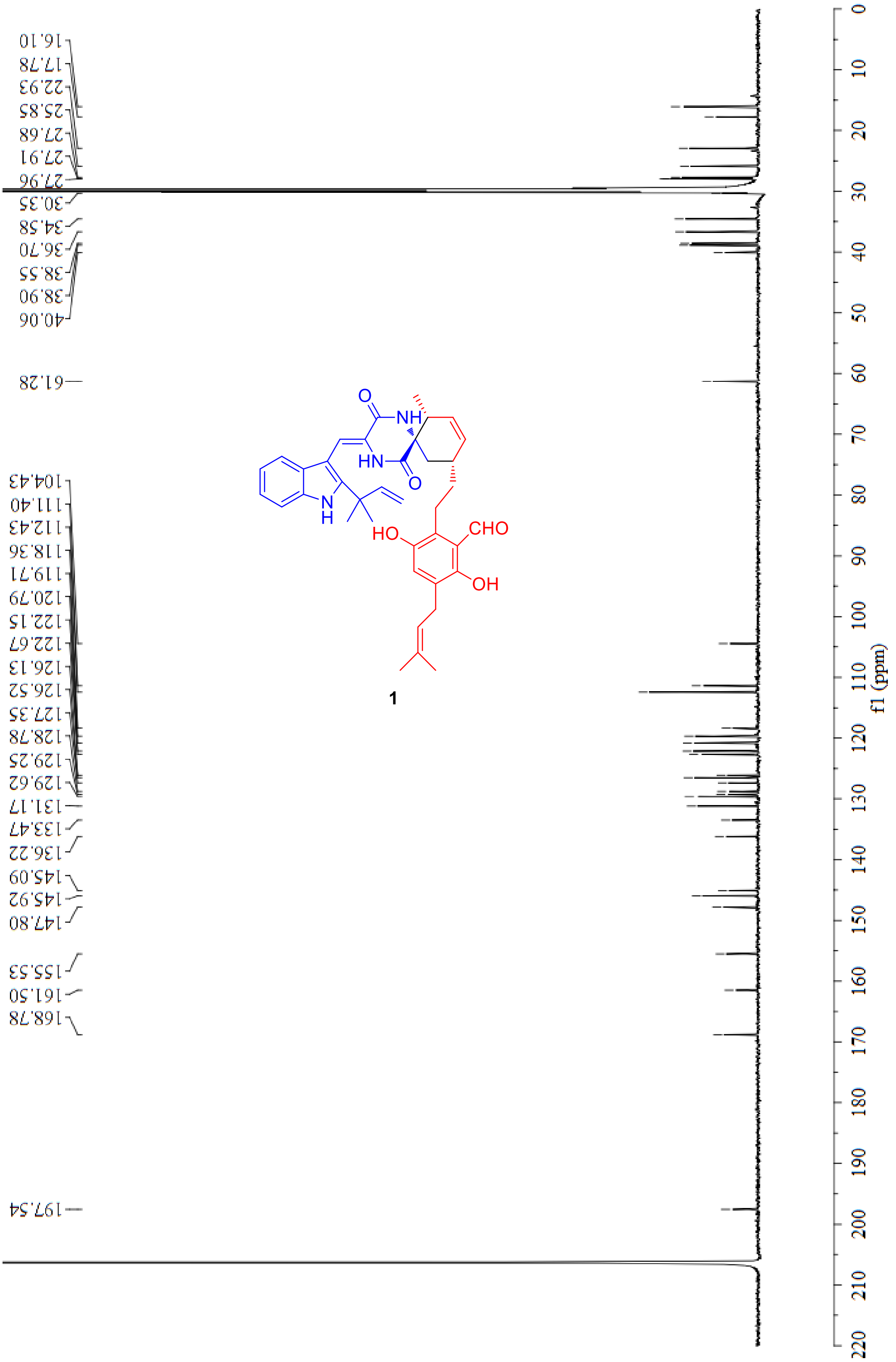

Figure S10 The HSQC spectrum of eurotinoid A (**1**) in CD_3COCD_3.

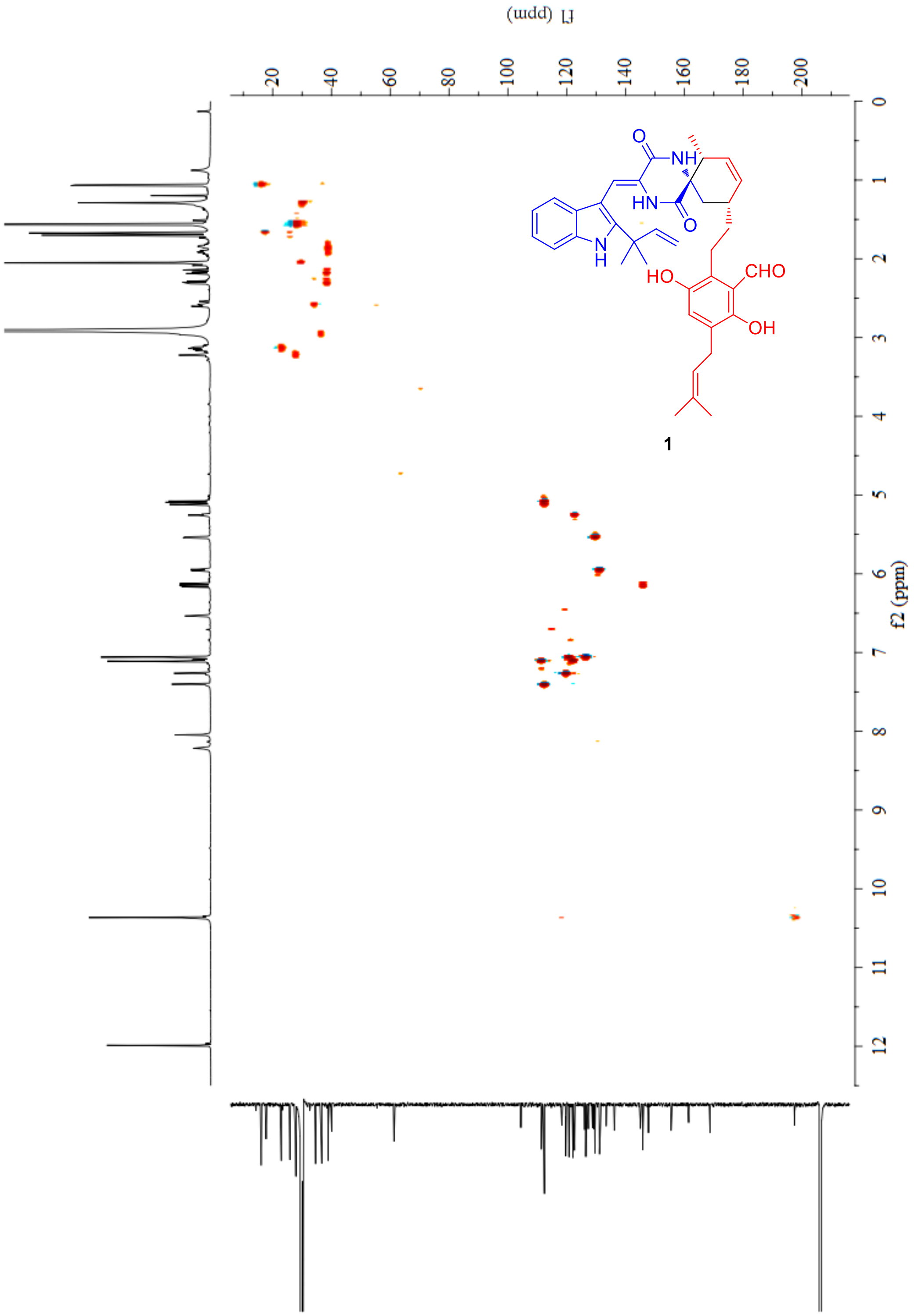

Figure S11 The HMBC spectrum of eurotinoid A (**1**) in CD_3COCD_3.

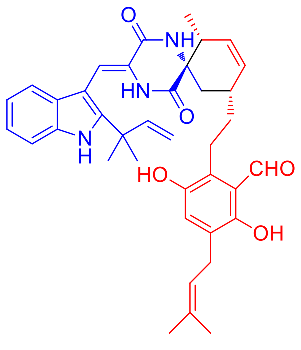

1

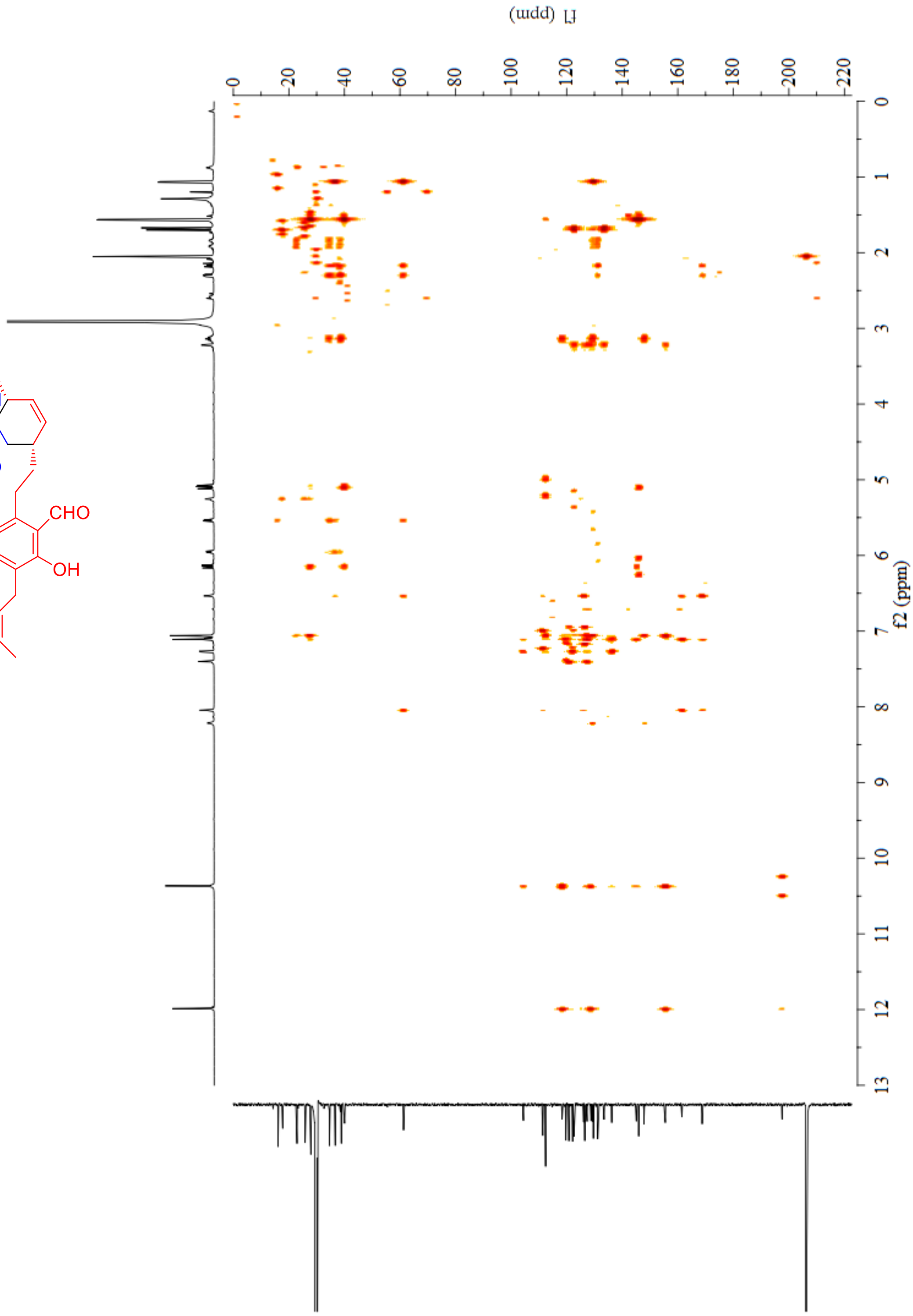

Figure S12 The ^{1}H–^{1}H COSY spectrum of eurotinoid A (**1**) in CD_3COCD_3.

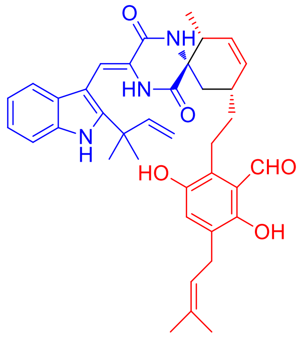

1

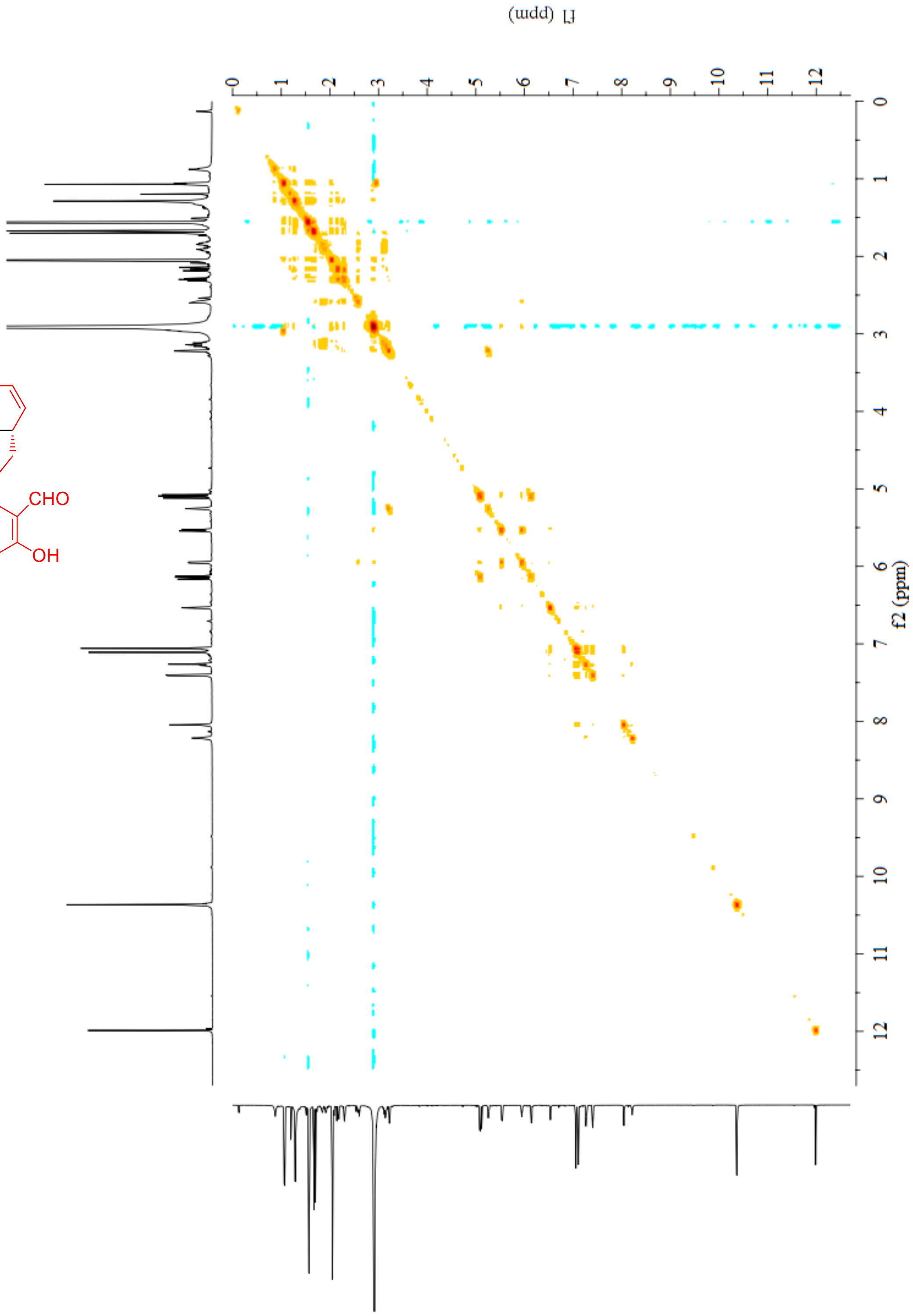

Figure S13 The NOESY spectrum of eurotinoid A (**1**) in CD_3COCD_3.

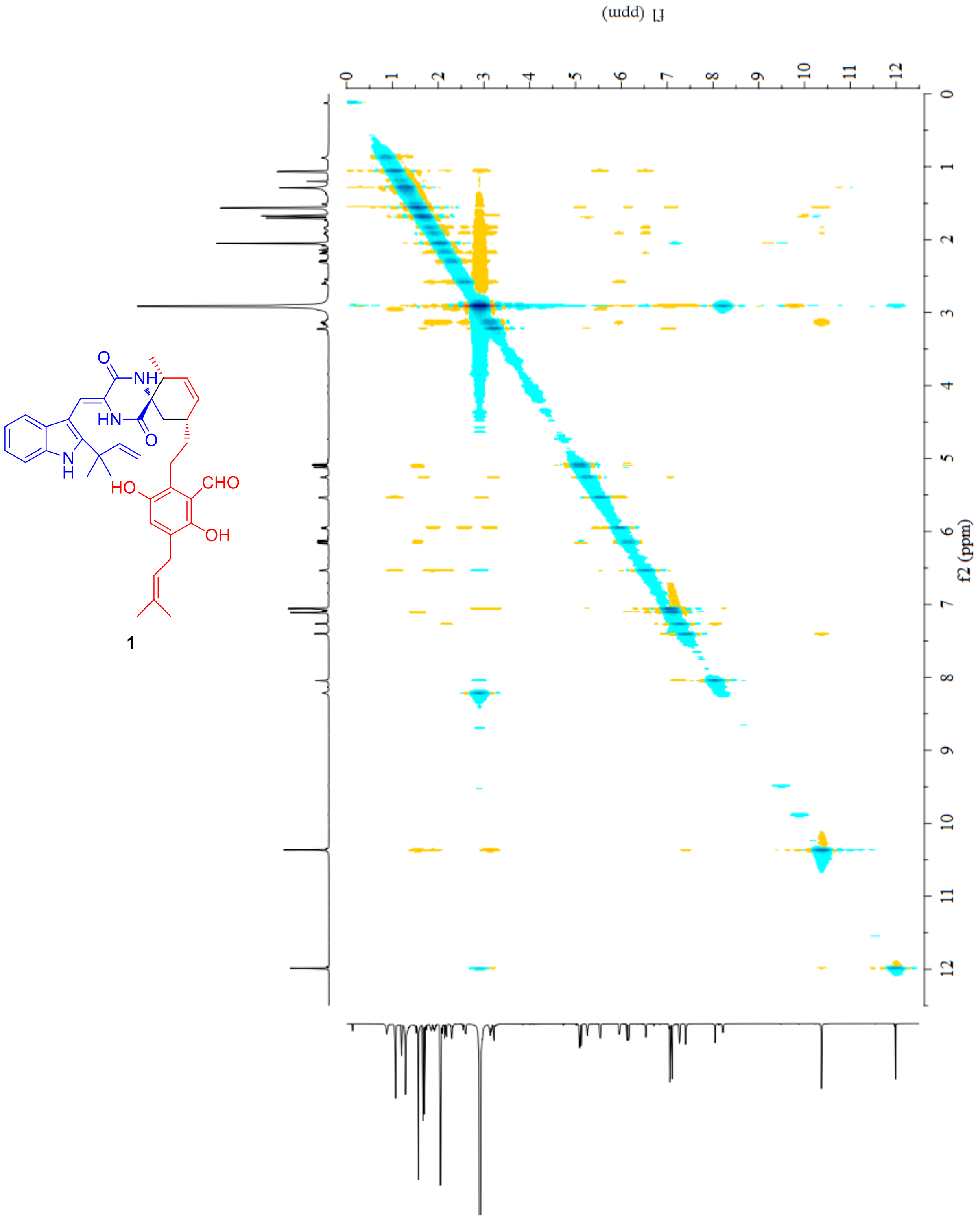

Figure S14 The HRESIMS spectrum of eurotinoid A (**1**) in CD_3COCD_3.

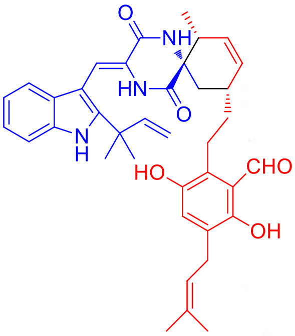

1

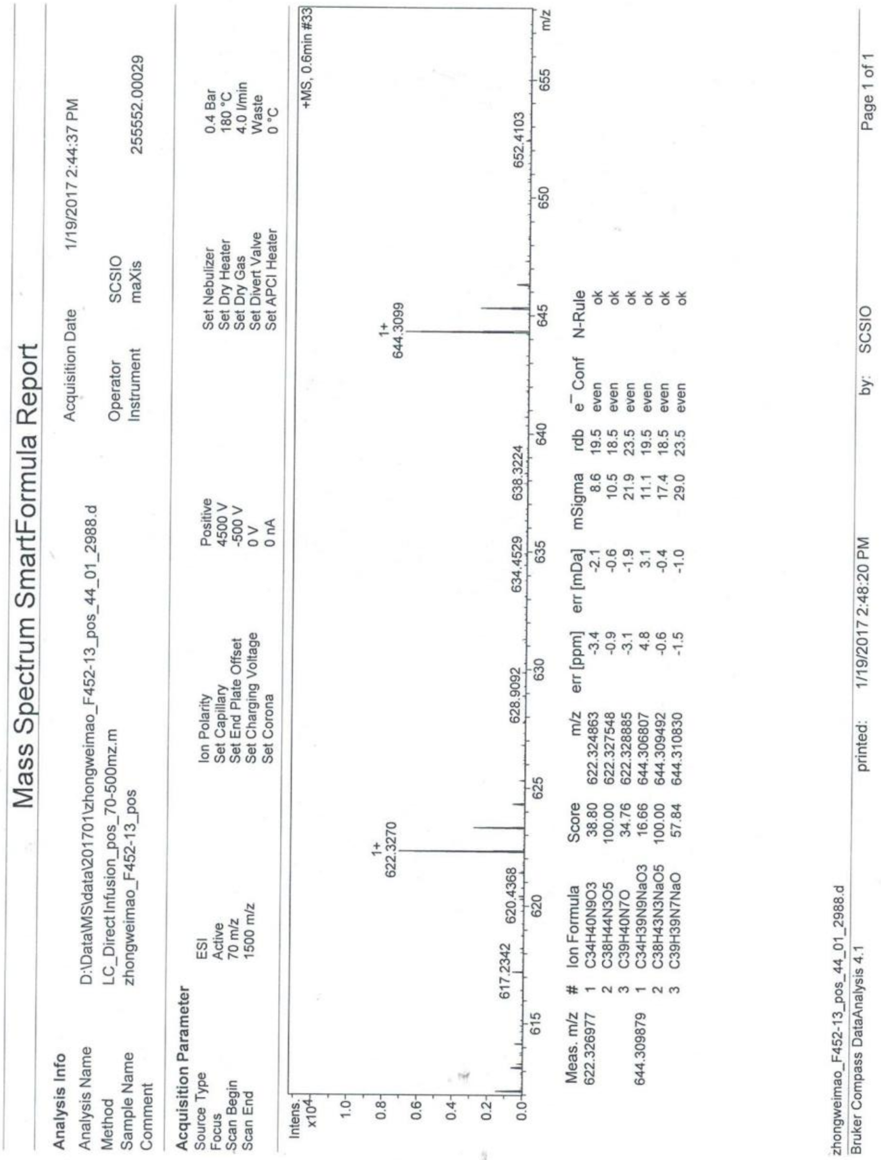

Mass Spectrum SmartFormula Report

Analysis Info

Analysis Name	D:\Data\MS\data\201701\zhongweimao_F452-13_pos_44_01_2988.d	Acquisition Date	1/19/2017 2:44:37 PM
Method	LC_Direct Infusion_pos_70-500mz.m	Operator	SCSIO
Sample Name	zhongweimao_F452-13_pos	Instrument	maXis 255552.00029
Comment			

Acquisition Parameter

Source Type	ESI	Ion Polarity	Positive	Set Nebulizer	0.4 Bar
Focus	Active	Set Capillary	4500 V	Set Dry Heater	180 °C
Scan Begin	70 m/z	Set End Plate Offset	-500 V	Set Dry Gas	4.0 l/min
Scan End	1500 m/z	Set Charging Voltage	0 V	Set Divert Valve	Waste
		Set Corona	0 nA	Set APCI Heater	0 °C

Meas. m/z	#	Ion Formula	Score	m/z	err [ppm]	err [mDa]	mSigma	rdb	e^- Conf	N-Rule
622.326977	1	C34H40N9O3	38.80	622.324863	-3.4	-2.1	8.6	19.5	even	ok
	2	C38H44N3O5	100.00	622.327548	-0.9	-0.6	10.5	18.5	even	ok
	3	C39H40N7O	34.76	622.328885	-3.1	-1.9	21.9	23.5	even	ok
644.309879	1	C34H39N9NaO3	16.66	644.306807	4.8	3.1	11.1	19.5	even	ok
	2	C38H43N3NaO5	100.00	644.309492	-0.6	-0.4	17.4	18.5	even	ok
	3	C39H39N7NaO	57.84	644.310830	-1.5	-1.0	29.0	23.5	even	ok

zhongweimao_F452-13_pos_44_01_2988.d
Bruker Compass DataAnalysis 4.1 printed: 1/19/2017 2:48:20 PM by: SCSIO Page 1 of 1

Figure S15 The IR spectrum of eurotinoid A (**1**) in CD_3COCD_3.

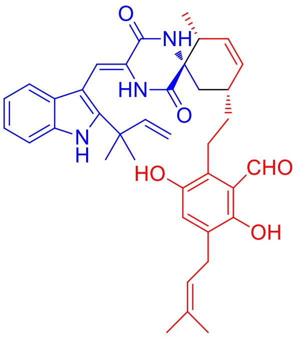

1

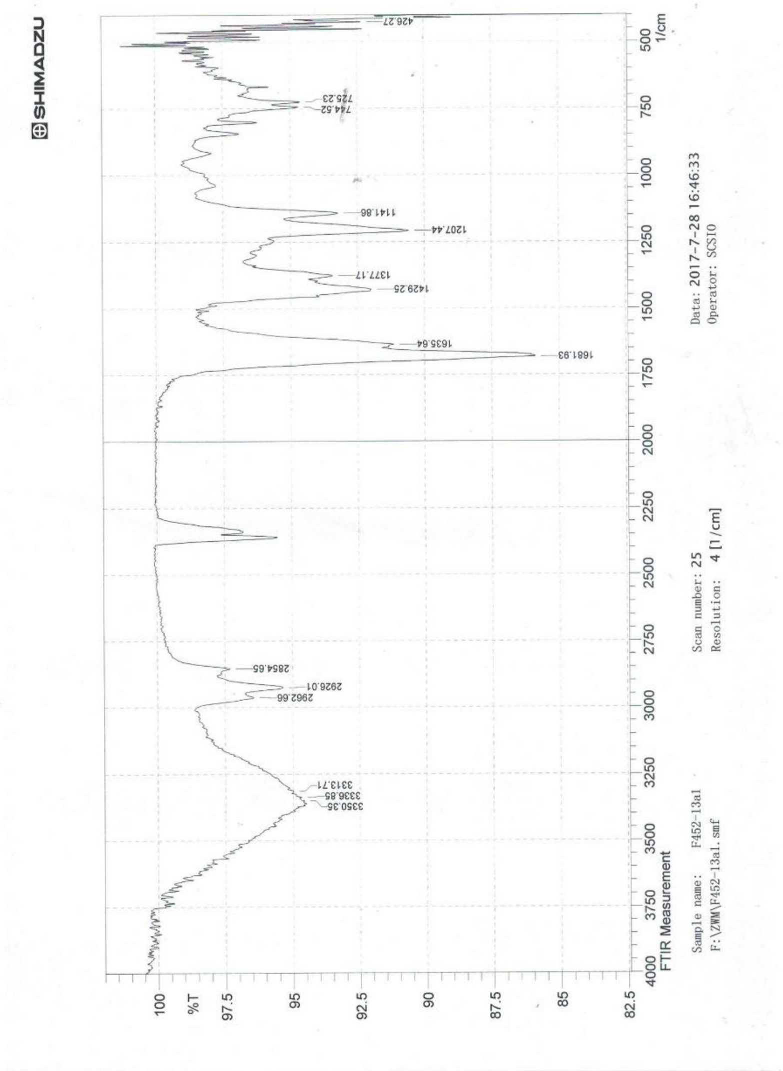

Figure S16 The UV spectrum of eurotinoid A (**1**) in CD_3COCD_3.

光谱峰值检测报告

2017-07-11 17:41:15

数据集: F452-13a-1-3 - RawData

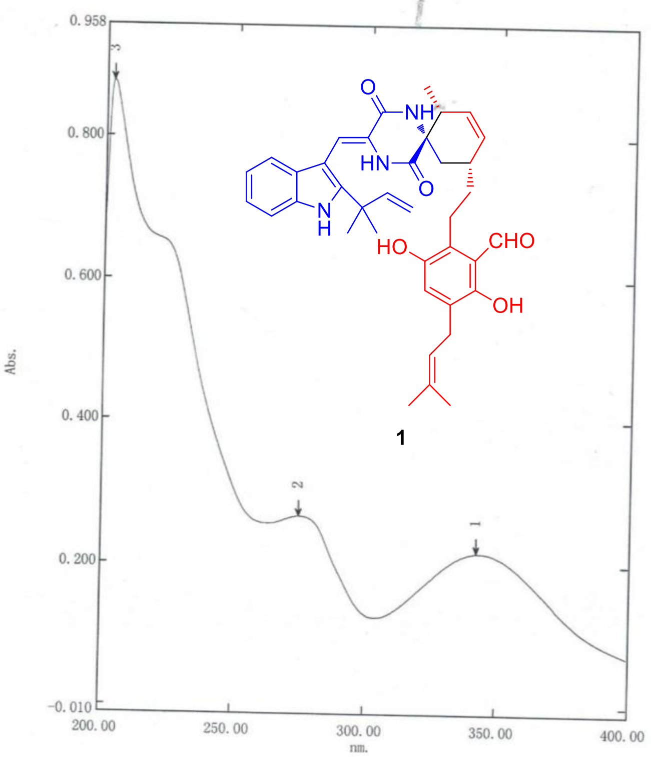

[测定属性]
波长范围 (nm.): 200.00 到 400.00
扫描速度: 中速
采样间隔: 0.2
自动采样间隔: 启用
扫描模式: 单个

[仪器属性]
仪器类型: UV-2600 系列
测定方式: 吸收值
狭缝宽: 2.0
积分时间: 0.1 秒.
光源转换波长: 323.0 nm
检测器单元: 直接
S/R 转换: 标准
阶梯校正: OFF

[附件属性]
附件: 无

[数据处理参数]
阈值: 0.0100000
点: 4
内插: 停用
平均: 停用

[样品准备属性]
重量:
体积:
稀释:
光程长:
附加信息:

No.	P/V	波长(nm)	吸收值	描述
1	↑	342.40	0.216	
2	↑	274.60	0.266	
3	↑	202.80	0.877	

页 1 / 1

Figure S17 The ^{1}H NMR spectrum of eurotinoid B (**2**) in CD_3COCD_3.

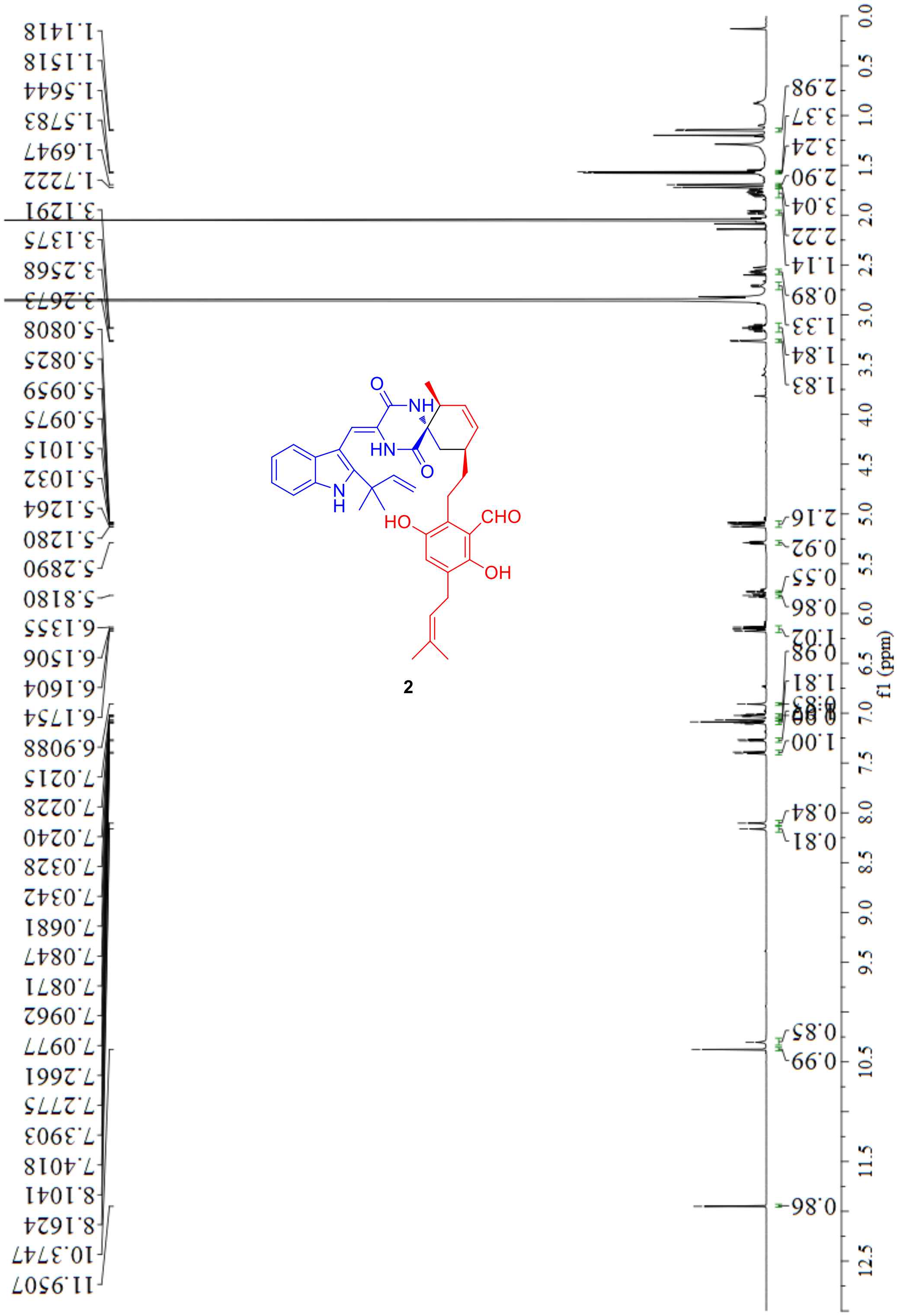

Figure S18 The ^{13}C NMR spectrum of eurotinoid B (**2**) in CD_3COCD_3.

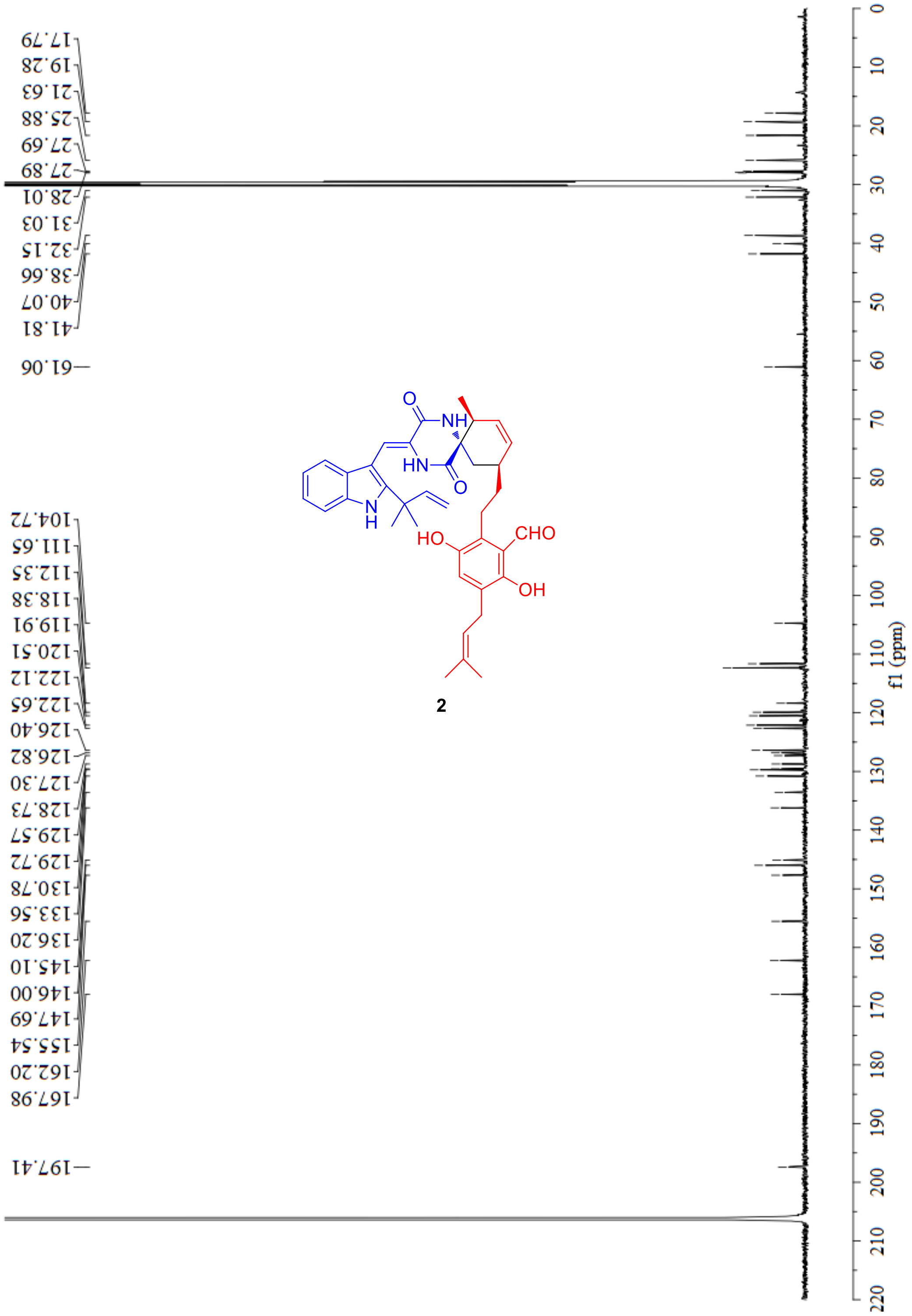

Figure S19 The HSQC spectrum of eurotinoid B (**2**) in CD_3COCD_3.

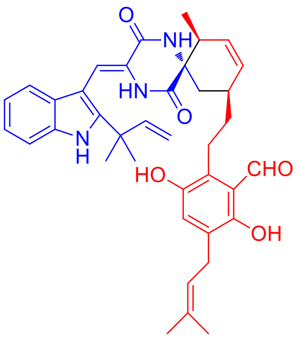

2

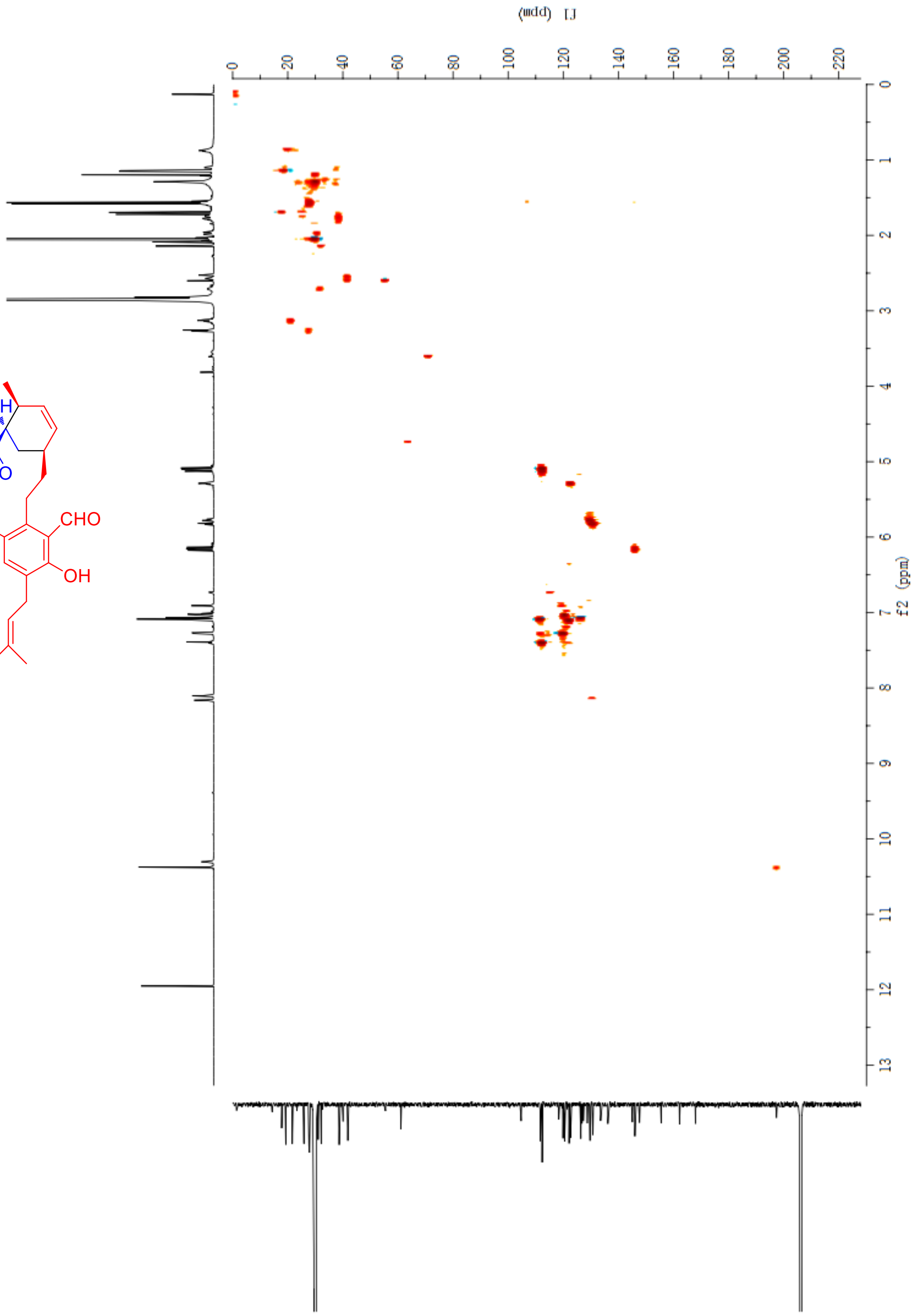

Figure S20 The HMBC spectrum of eurotinoid B (**2**) in CD_3COCD_3.

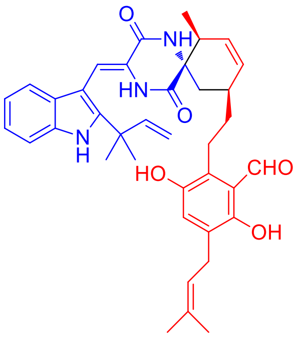

2

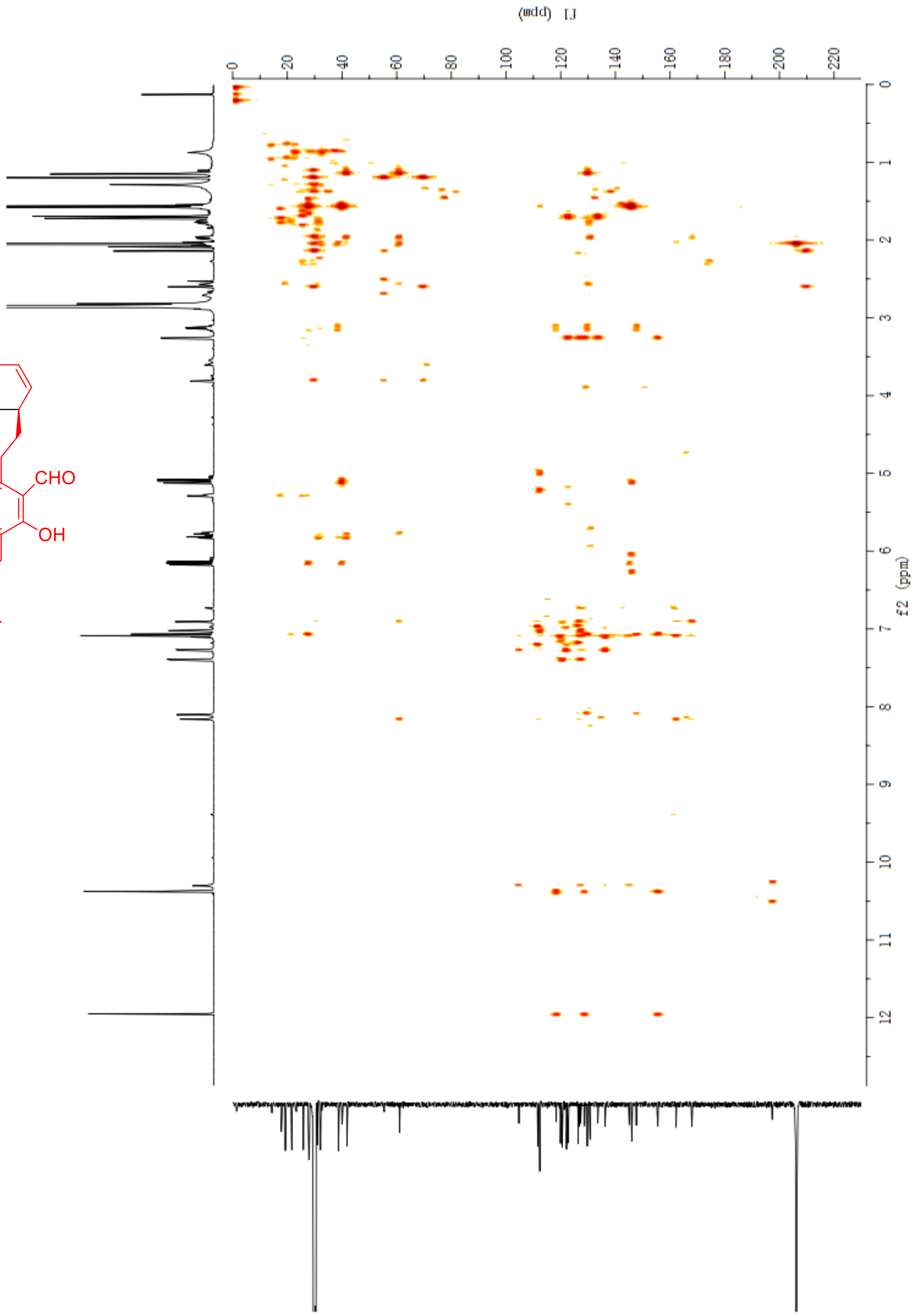

Figure S21 The ^1H–^1H COSY spectrum of eurotinoid B (**2**) in CD_3COCD_3.

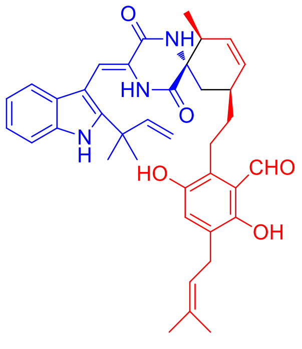

2

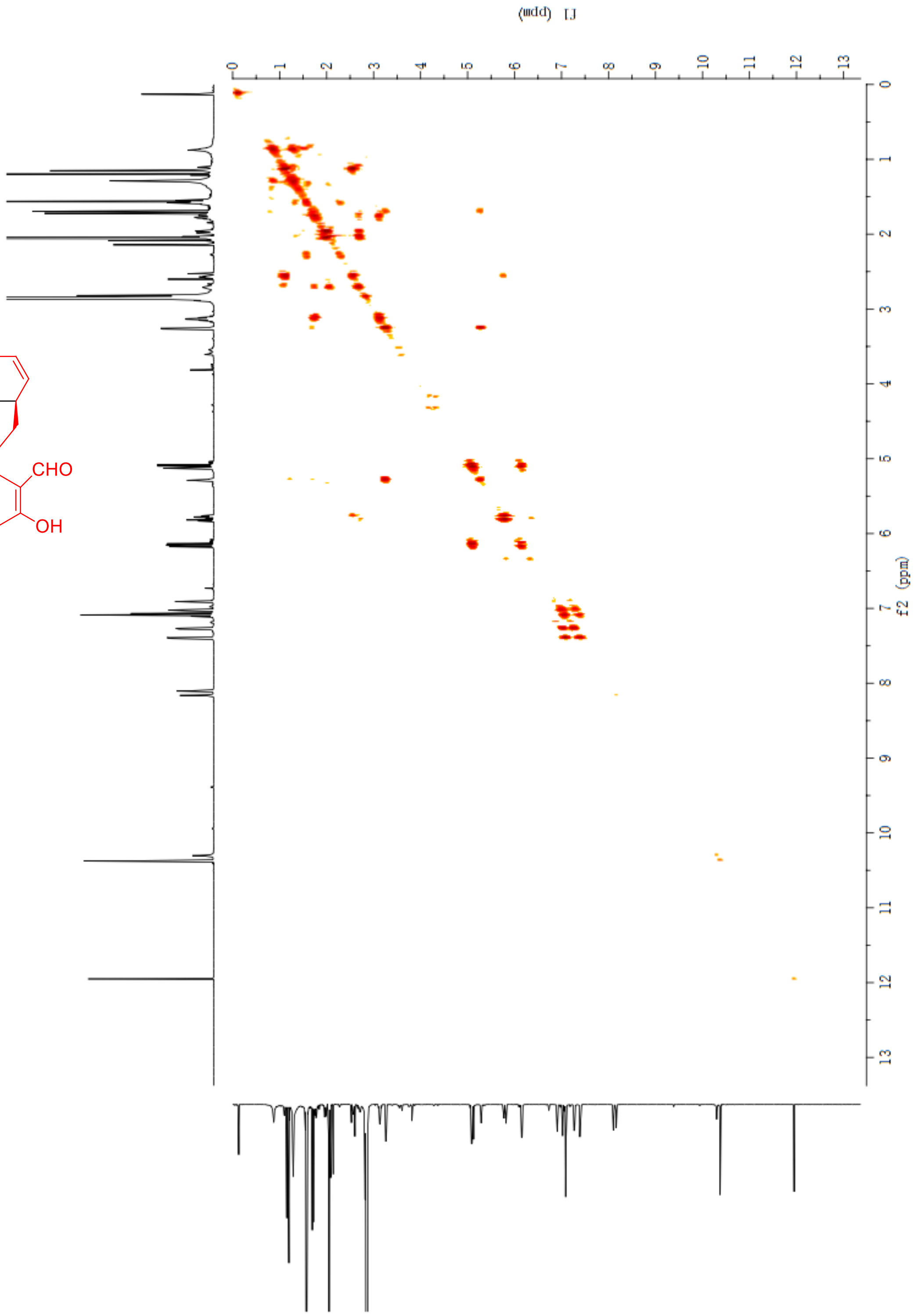

Figure S22 The NOESY spectrum of eurotinoid B (**2**) in CD_3COCD_3.

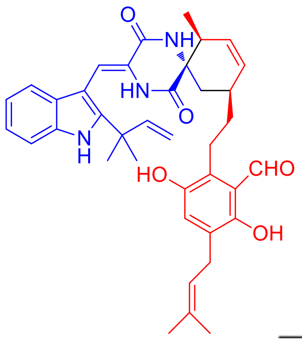

2

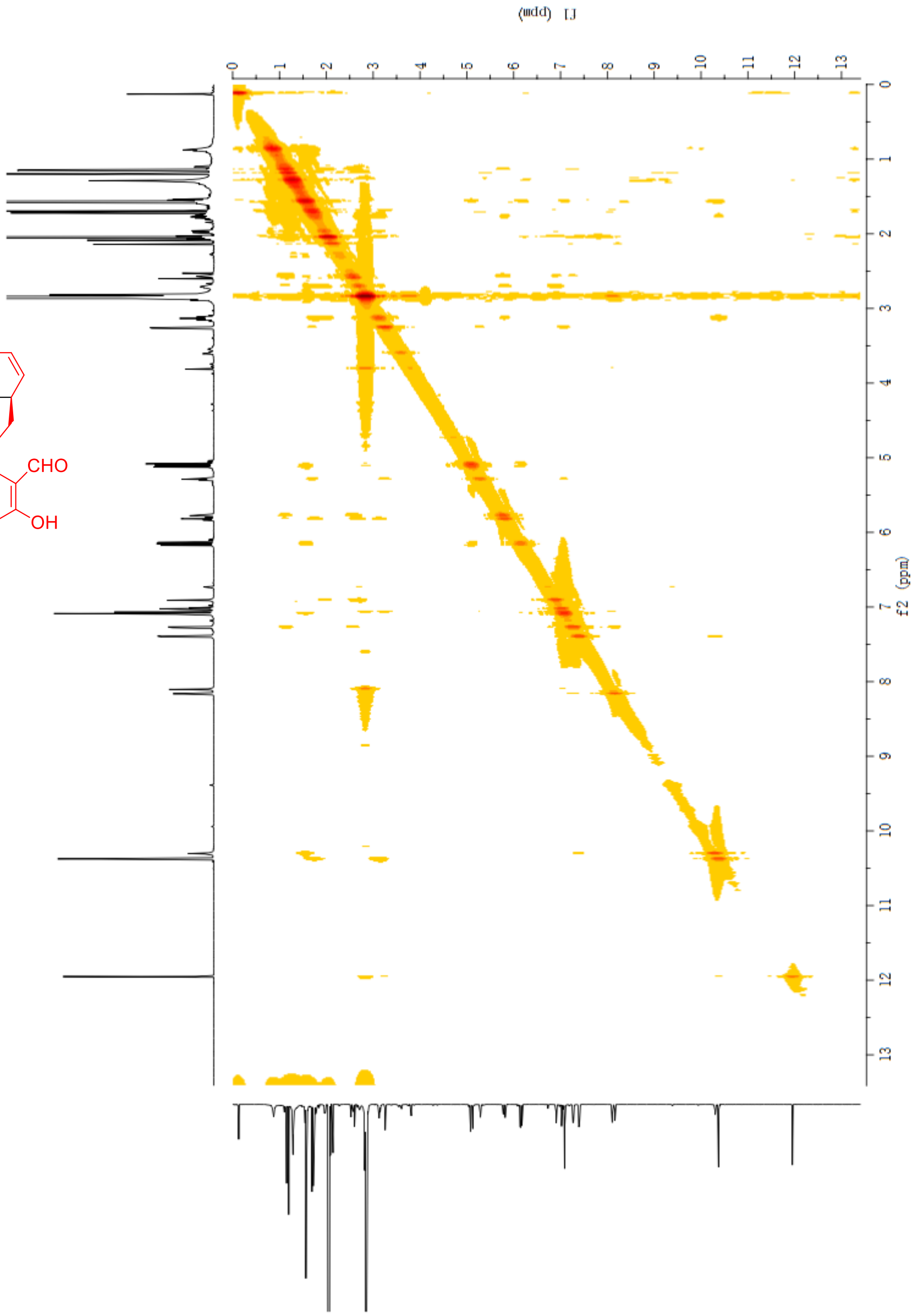

Figure S23 The HRESIMS spectrum of eurotinoid B (**2**).

Mass Spectrum SmartFormula Report

Analysis Info

Analysis Name	D:\Data\MS\data\201707\zhongweimao_F425-52_pos_36_01_3290.d	Acquisition Date	7/26/2017 4:51:54 PM
Method	LC_Direct Infusion_pos_70-500mz.m	Operator	SCSIO
Sample Name	zhongweimao_F425-52_pos	Instrument	maXis 255552.00029
Comment			

Acquisition Parameter

Source Type	ESI	Ion Polarity	Positive	Set Nebulizer	0.4 Bar
Focus	Active	Set Capillary	4500 V	Set Dry Heater	180 °C
Scan Begin	70 m/z	Set End Plate Offset	-500 V	Set Dry Gas	4.0 l/min
Scan End	1500 m/z	Set Charging Voltage	0 V	Set Divert Valve	Waste
		Set Corona	0 nA	Set APCI Heater	0 °C

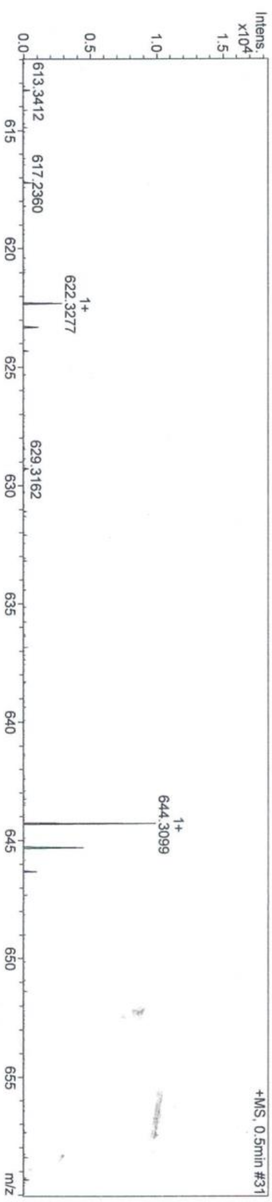

Meas. m/z	#	Ion Formula	Score	m/z	err [ppm]	err [mDa]	mSigma	rdb	e⁻ Conf	N-Rule
622.327705	1	$C_{38}H_{44}N_3O_5$	100.00	622.327548	-0.3	-0.2	25.4	18.5	even	ok
644.309927	1	$C_{38}H_{43}N_3NaO_5$	100.00	644.309492	-0.7	-0.4	15.4	18.5	even	ok
1243.648761	1	$C_{76}H_{87}N_6O_{10}$	100.00	1243.647819	0.8	0.9	60.1	36.5	even	ok
1265.630677	1	$C_{76}H_{86}N_6NaO_{10}$	100.00	1265.629764	-0.7	-0.9	39.6	36.5	even	ok

zhongweimao_F425-52_pos_36_01_3290.d

Bruker Compass DataAnalysis 4.1 printed: 7/26/2017 5:05:36 PM by: SCSIO Page 1 of 1

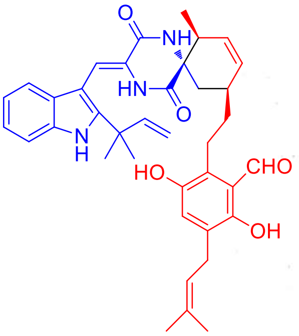

2

Figure S24 The UV spectrum of eurotinoid B (**2**).

光谱选点检测报告

2018-01-04 18:21:08

数据集: F452-52-1-5 - RawData

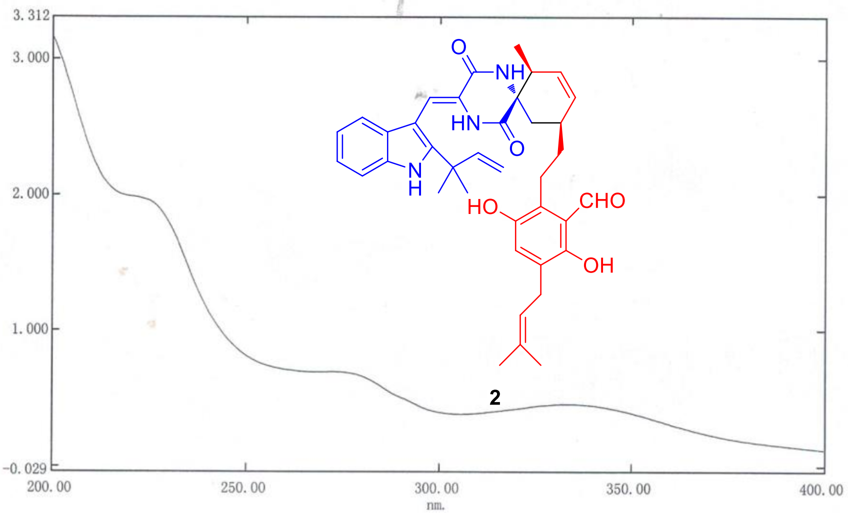

[测定属性]
波长范围 (nm.): 200. 00 到 400. 00
扫描速度: 中速
采样间隔: 0. 2
自动采样间隔: 启用
扫描模式: 单个

No.	波长(nm)	吸收值	描述
1	333. 60	0. 461	
2	276. 40	0. 690	
3	225. 20	1. 956	
4			

[仪器属性]
仪器类型: UV-2600 系列
测定方式: 吸收值
狭缝宽: 2. 0
积分时间: 0. 1 秒.
光源转换波长: 323. 0 nm
检测器单元: 直接
S/R 转换: 标准
阶梯校正: OFF

[附件属性]
附件: 无

[数据处理参数]
阈值: 0. 0100000
点: 4
内插: 停用
平均: 停用

[样品准备属性]
重量:
体积:
稀释:
光程长:
附加信息:

页 1 / 1

Figure S25 The ^{1}H NMR spectrum of eurotinoid C (**3**) in DMSO-d_6.

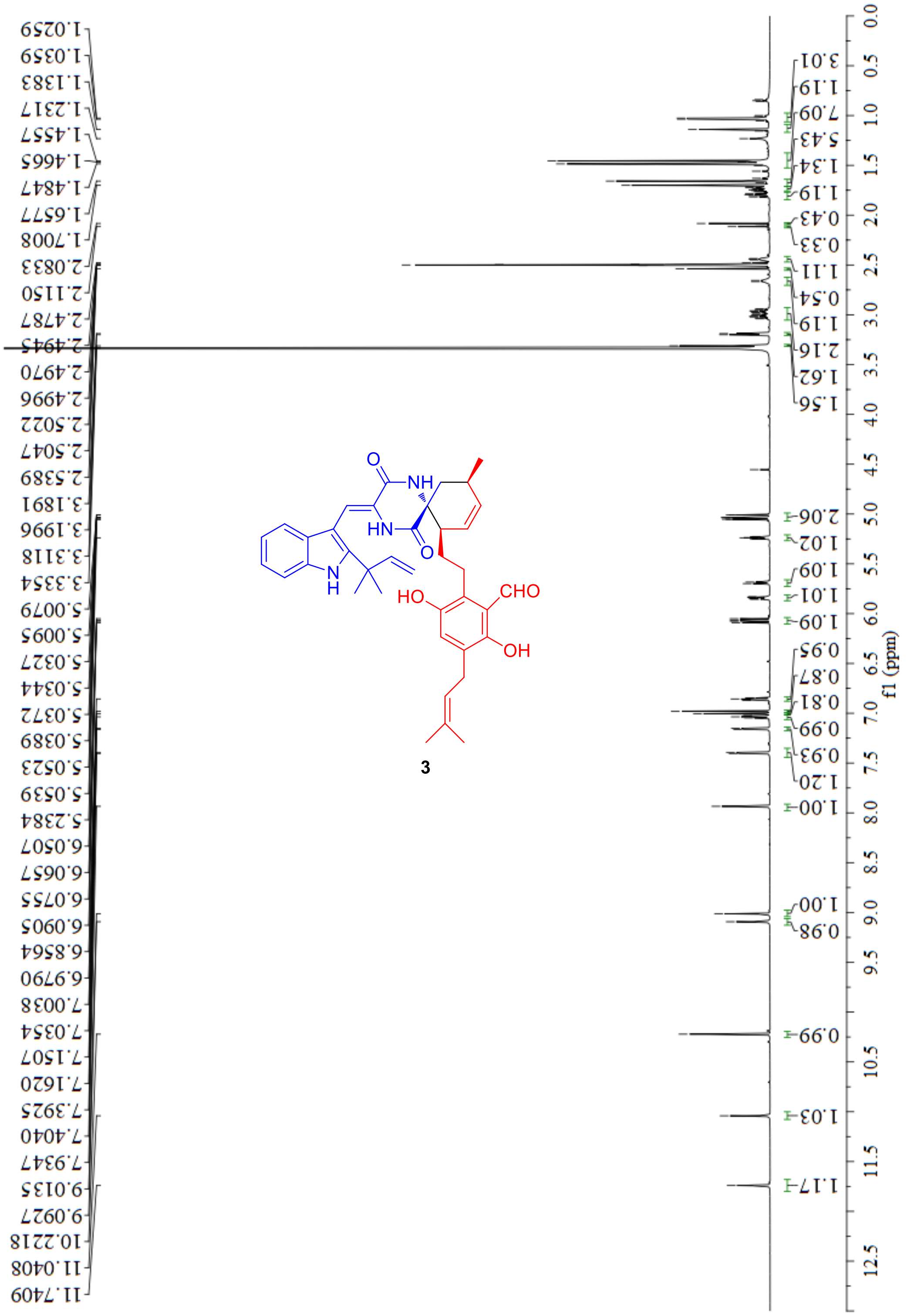

Figure S26 The ^{13}C NMR spectrum of eurotinoid C (**3**) in DMSO-d_6.

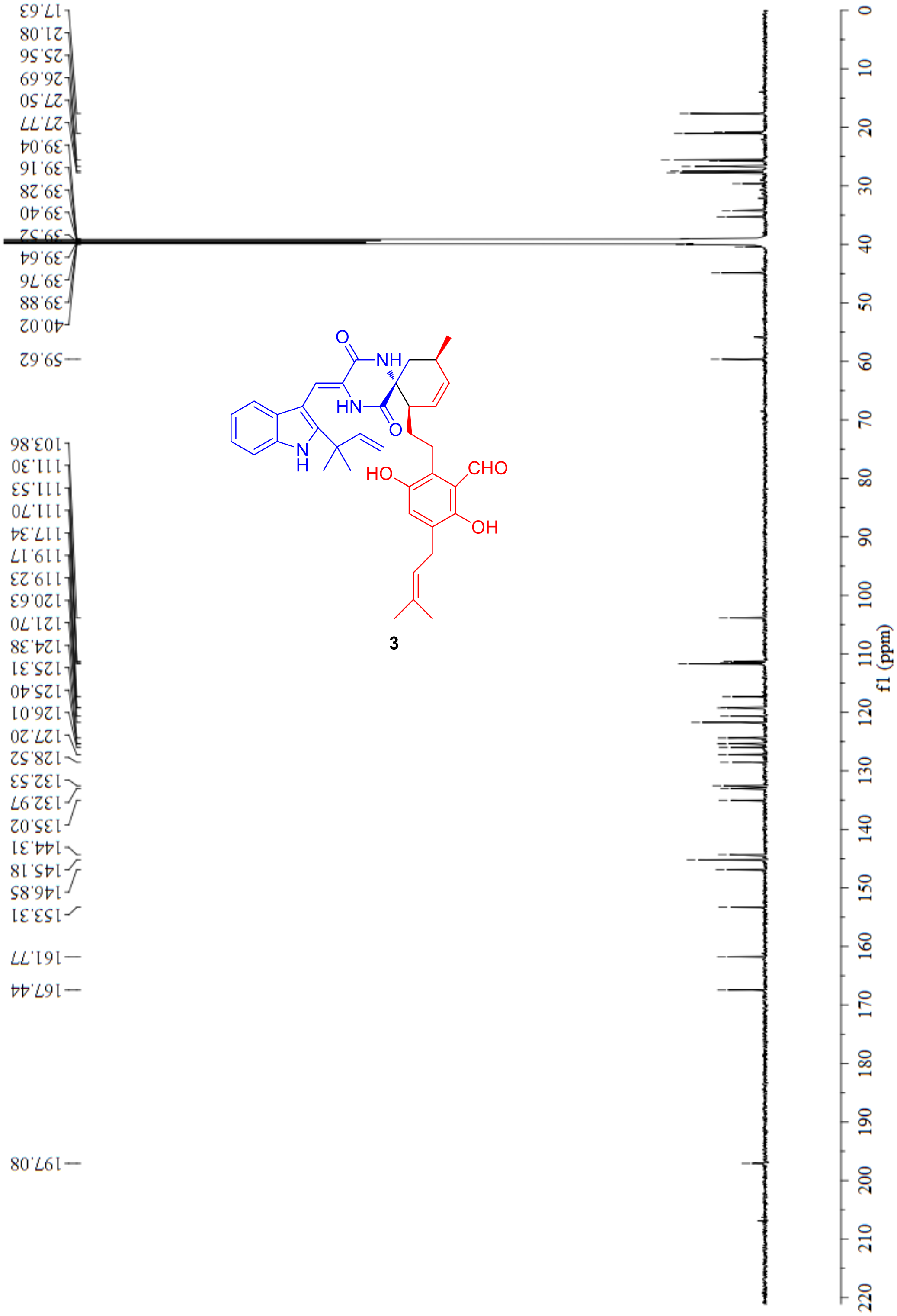

Figure S27 The HSQC spectrum of eurotinoid C (**3**) in DMSO-d_6.

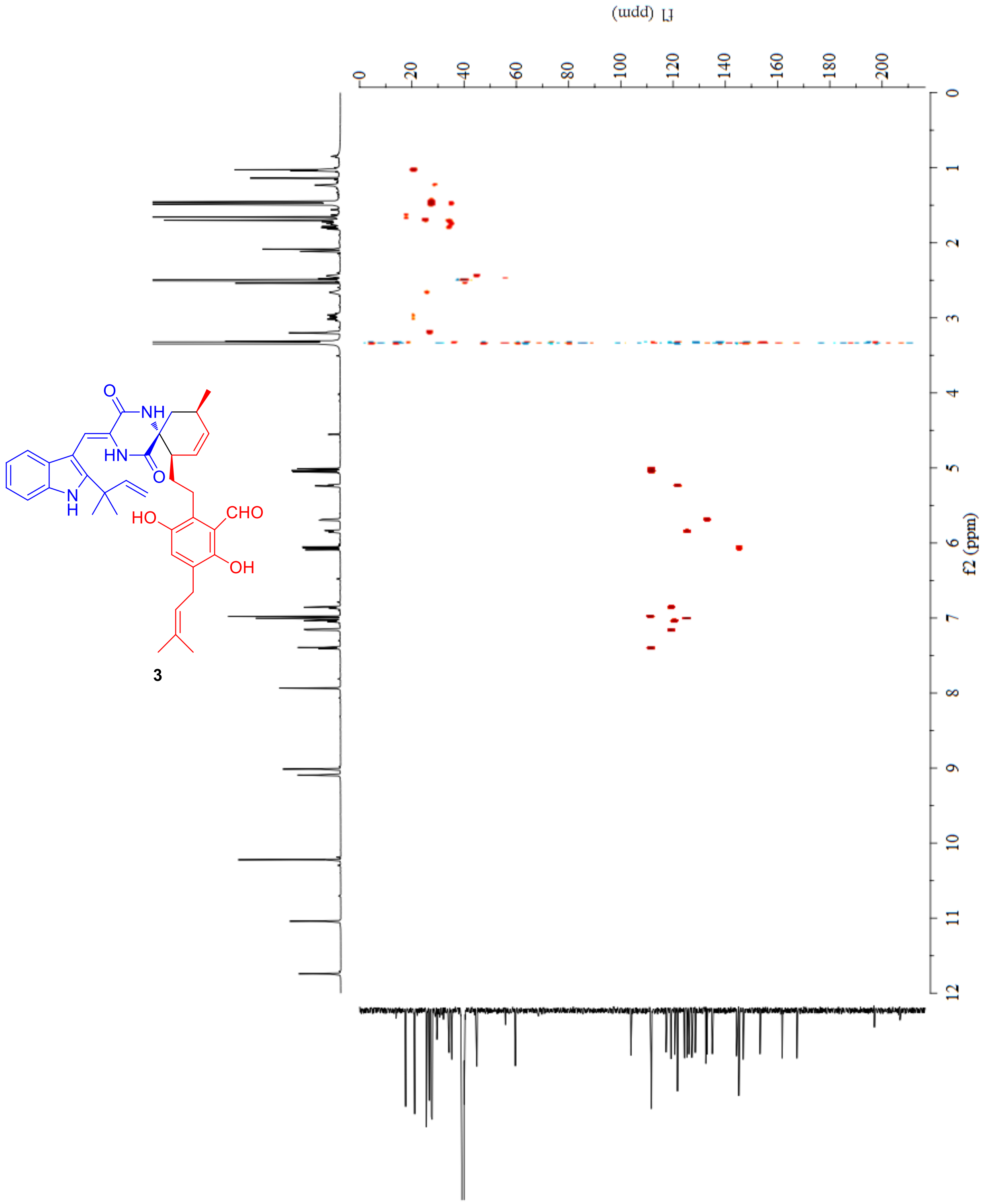

Figure S28 The HMBC spectrum of eurotinoid C (**3**) in DMSO-d_6.

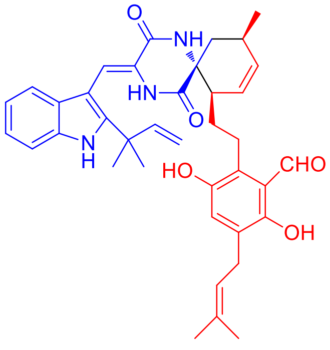

3

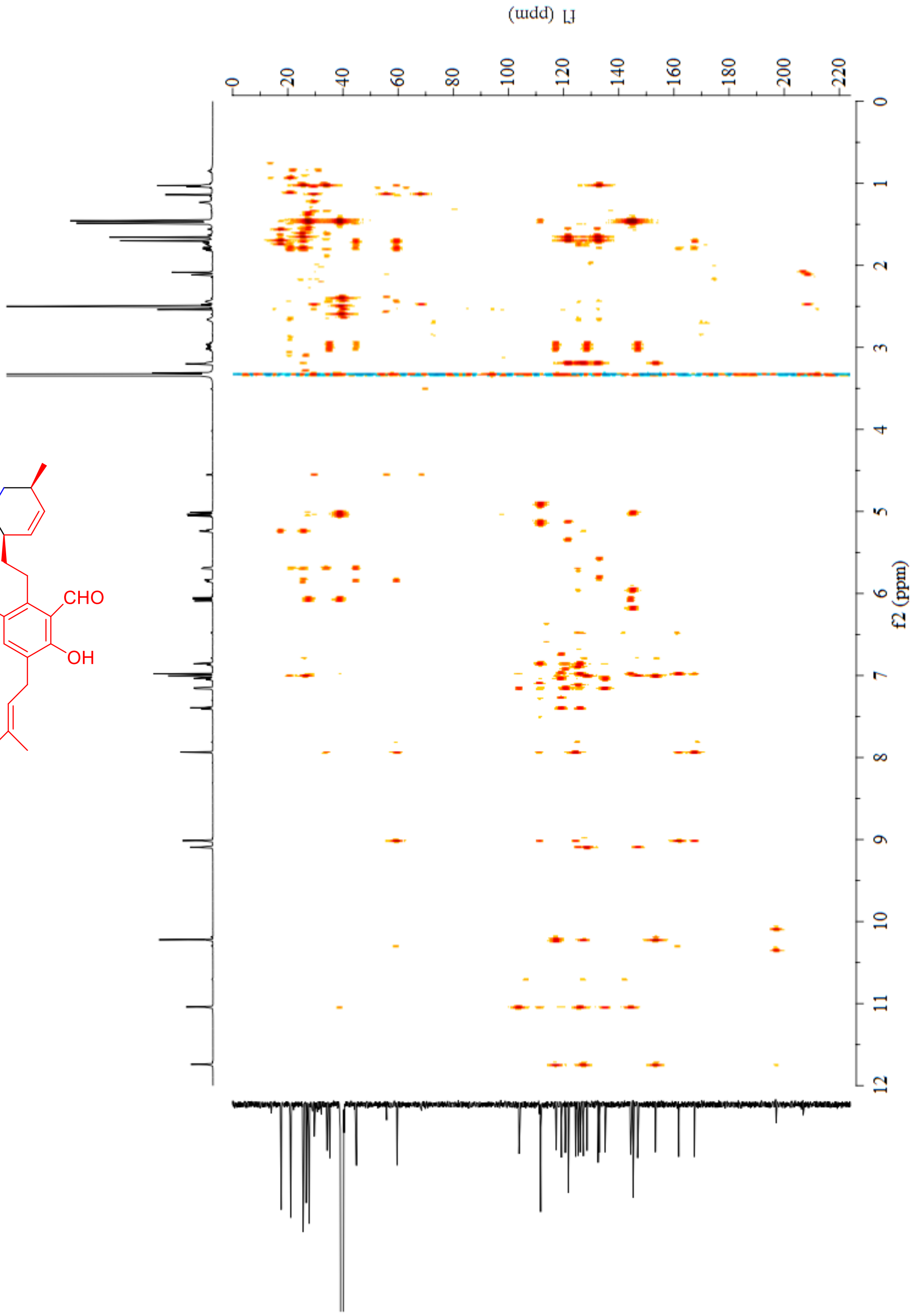

Figure S29 The ^1H–^1H COSY spectrum of eurotinoid C (**3**) in DMSO-d_6.

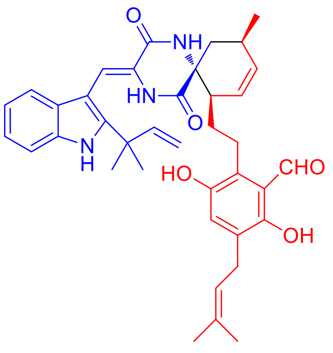

3

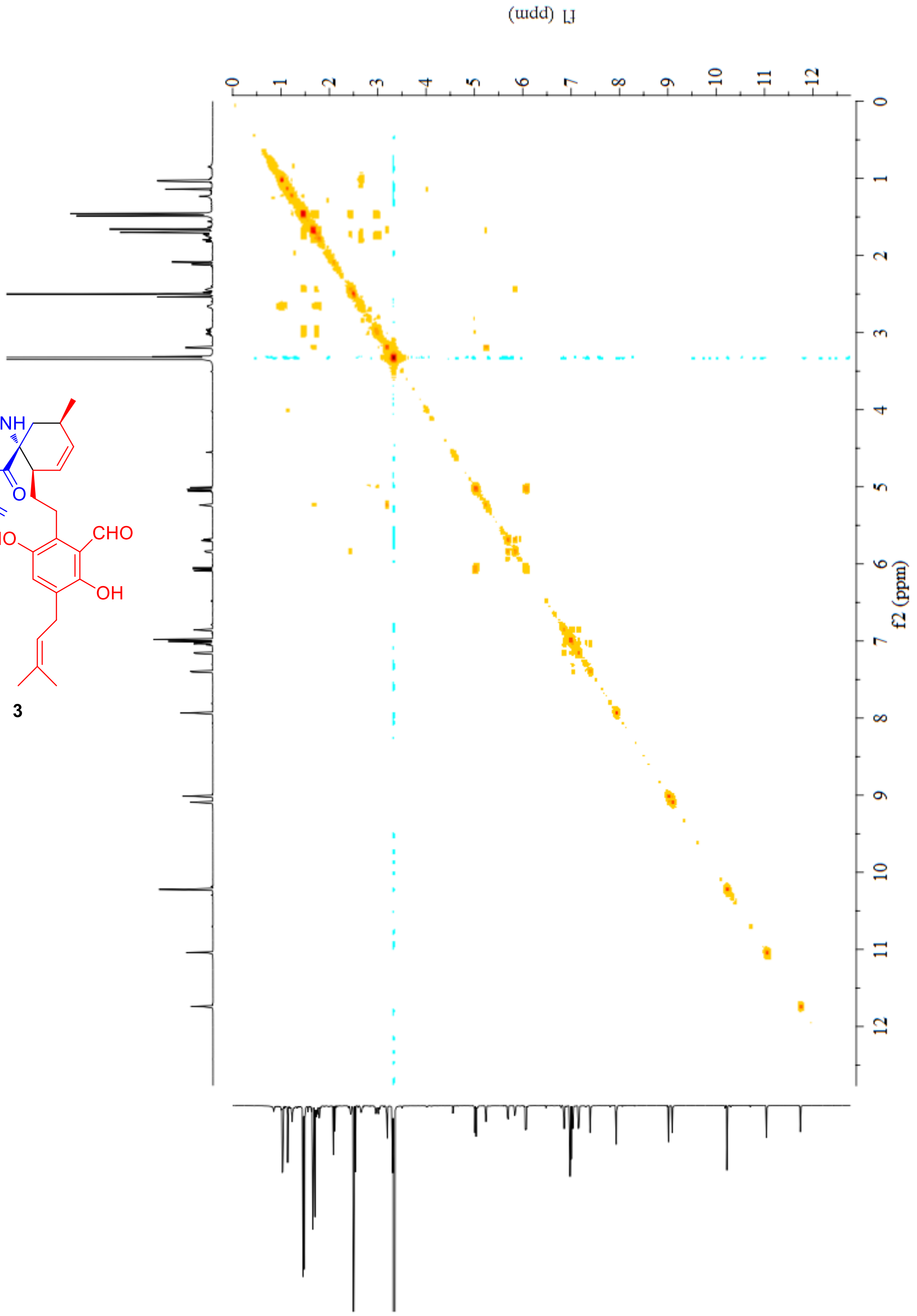

Figure S30 The NOESY spectrum of eurotinoid C (**3**) in DMSO-d_6.

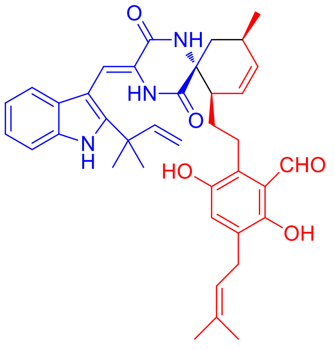

3

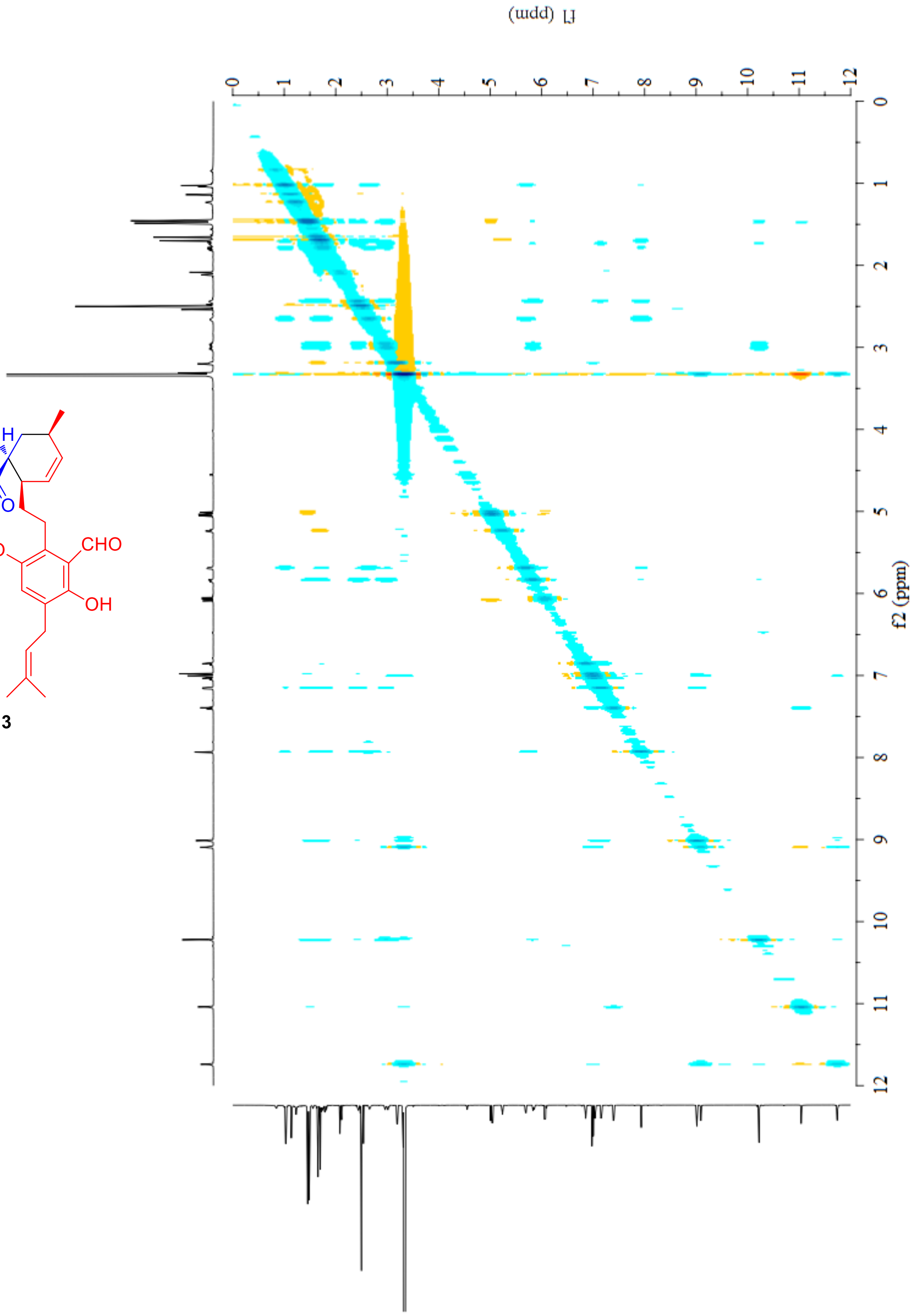

Figure S31 The HRESIMS spectrum of eurotinoid C (**3**).

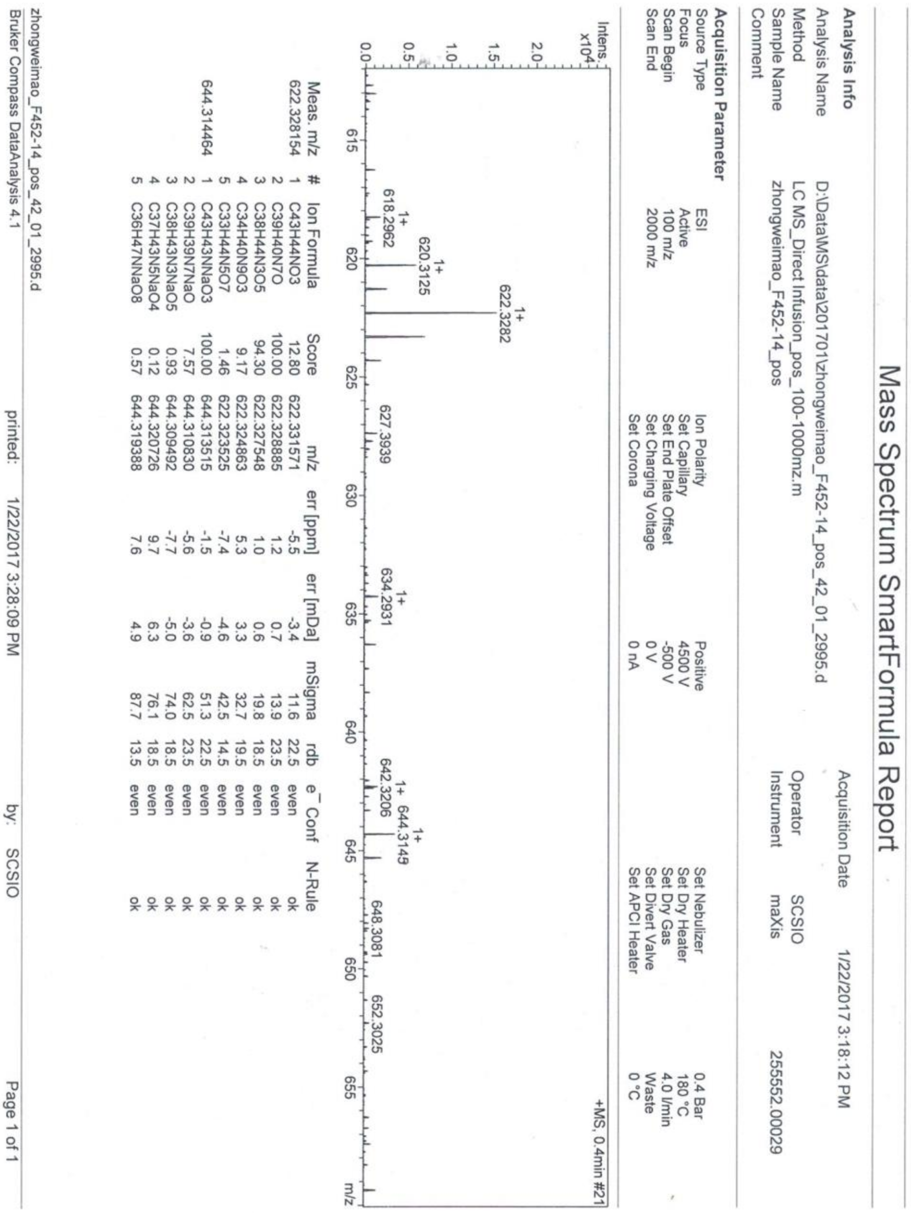

Mass Spectrum SmartFormula Report

Analysis Info

Analysis Name: D:\Data\MS\data\201701\zhongweimao_F452-14_pos_42_01_2995.d
Method: LC MS_Direct Infusion_pos_100-1000mz.m
Sample Name: zhongweimao_F452-14_pos
Comment:

Acquisition Date: 1/22/2017 3:18:12 PM
Operator: SCSIO
Instrument: maXis 255552.00029

Acquisition Parameter

Source Type	ESI	Ion Polarity	Positive	Set Nebulizer	0.4 Bar
Focus	Active	Set Capillary	4500 V	Set Dry Heater	180 °C
Scan Begin	100 m/z	Set End Plate Offset	-500 V	Set Dry Gas	4.0 l/min
Scan End	2000 m/z	Set Charging Voltage	0 V	Set Divert Valve	Waste
		Set Corona	0 nA	Set APCI Heater	0 °C

Meas. m/z	#	Ion Formula	Score	m/z	err [ppm]	err [mDa]	mSigma	rdb	e⁻ Conf	N-Rule
622.328154	1	C43H44NO3	12.80	622.331571	-5.5	-3.4	11.6	22.5	even	ok
	2	C39H40N7O	100.00	622.328885	1.2	0.7	13.9	23.5	even	ok
	3	C38H44N3O5	94.30	622.327548	1.0	0.6	19.8	18.5	even	ok
	4	C34H40N9O3	9.17	622.324863	5.3	3.3	32.7	19.5	even	ok
	5	C33H44N5O7	1.46	622.323525	-7.4	-4.6	42.5	14.5	even	ok
644.314464	1	C43H43NNaO3	100.00	644.313515	-1.5	-0.9	51.3	22.5	even	ok
	2	C39H39N7NaO	7.57	644.310830	-5.6	-3.6	62.5	23.5	even	ok
	3	C38H43N3NaO5	0.93	644.309492	-7.7	-5.0	74.0	18.5	even	ok
	4	C37H43N5NaO4	0.12	644.320726	9.7	6.3	76.1	18.5	even	ok
	5	C36H47NNaO8	0.57	644.319388	7.6	4.9	87.7	13.5	even	ok

zhongweimao_F452-14_pos_42_01_2995.d
Bruker Compass DataAnalysis 4.1 printed: 1/22/2017 3:28:09 PM by: SCSIO Page 1 of 1

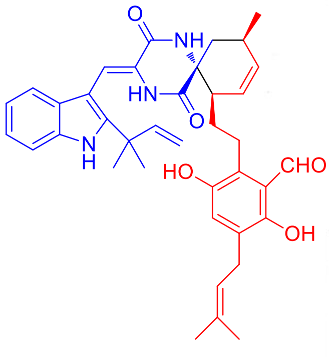

3

Figure S32 The IR spectrum of eurotinoid C (**3**).

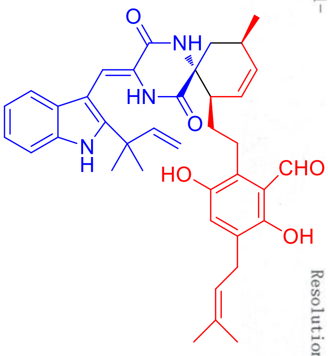

3

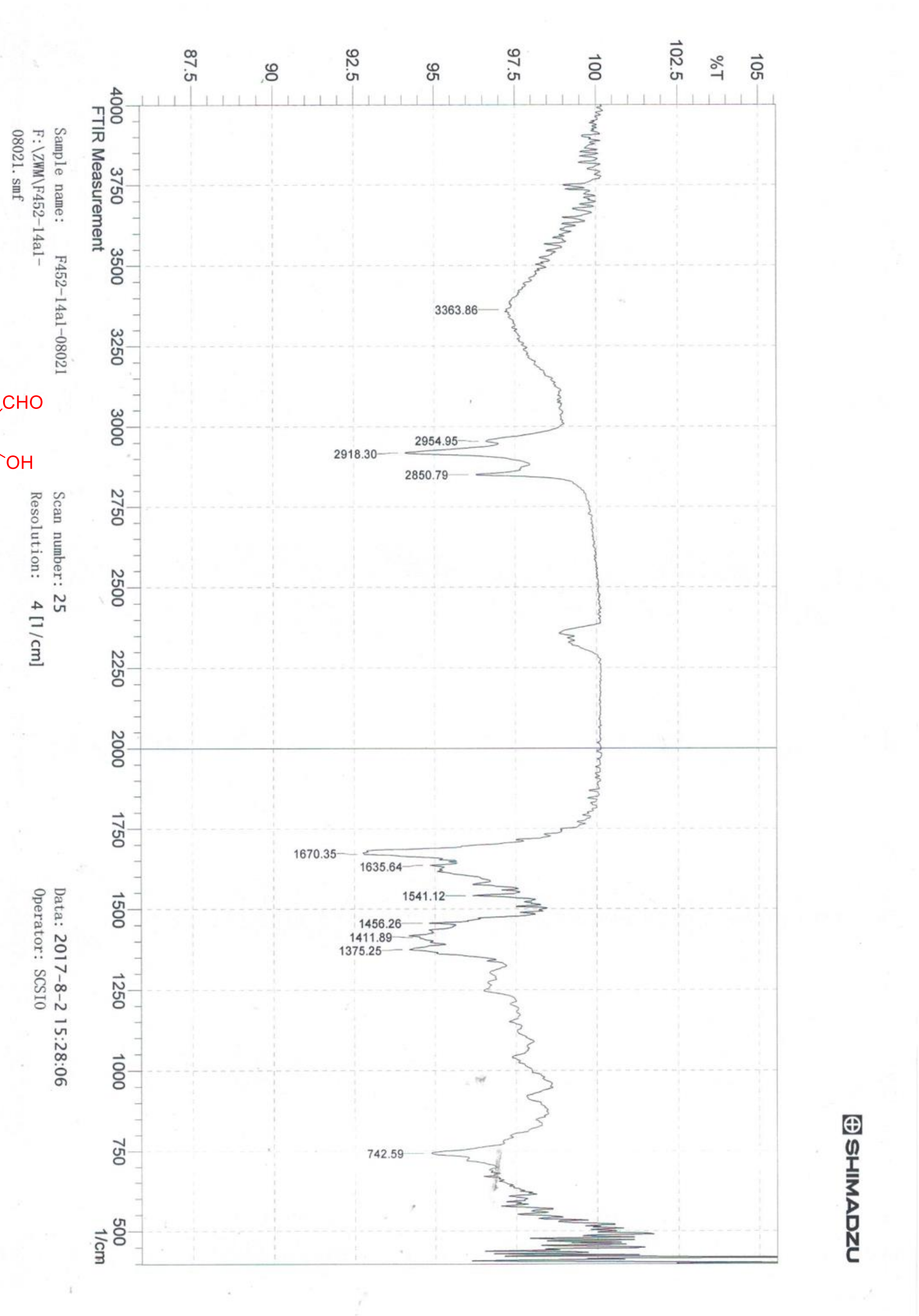

Figure S33 The UV spectrum of eurotinoid C (**3**).

光谱峰值检测报告

2017-07-11 17:34:21

数据集: F452-14a-1 - RawData

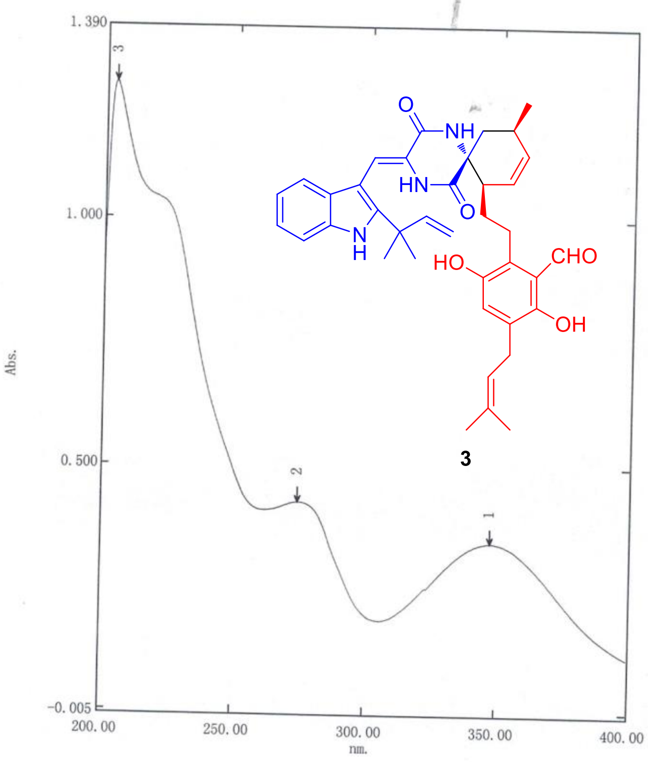

[测定属性]
波长范围 (nm.): 200.00 到 400.00
扫描速度: 中速
采样间隔: 0.2
自动采样间隔: 启用
扫描模式: 单个

[仪器属性]
仪器类型: UV-2600 系列
测定方式: 吸收值
狭缝宽: 2.0
积分时间: 0.1 秒.
光源转换波长: 323.0 nm
检测器单元: 直接
S/R 转换: 标准
阶梯校正: OFF

[附件属性]
附件: 无

[数据处理参数]
阈值: 0.0100000
点: 4
内插: 停用
平均: 停用

[样品准备属性]
重量:
体积:
稀释:
光程长:
附加信息:

No.	P/V	波长(nm)	吸收值	描述
1	⬆	347.40	0.345	
2	⬆	274.40	0.425	
3	⬆	203.40	1.274	

页 1 / 1
